# Activation of apoptosis by rationally constructing NIR amphiphilic AIEgens: surmounting the shackle of mitochondrial membrane potential for amplified tumor ablation†

**DOI:** 10.1039/d1sc02227j

**Published:** 2021-07-06

**Authors:** Haidong Li, Yang Lu, Jeewon Chung, Jingjing Han, Heejeong Kim, Qichao Yao, Gyoungmi Kim, Xiaofeng Wu, Saran Long, Xiaojun Peng, Juyoung Yoon

**Affiliations:** Department of Chemistry and Nanoscience, Ewha Womans University Seoul 03760 Korea jyoon@ewha.ac.kr; State Key Laboratory of Fine Chemicals, Dalian University of Technology 2 Linggong Road Dalian 116024 P. R. China pengxj@dlut.edu.cn; Ningbo Institute of Dalian University of Technology 26 Yucai Road, Jiangbei District Ningbo 315016 P. R. China

## Abstract

In recent years, the use of aggregation-induced emission luminogens (AIEgens) for biological imaging and phototherapy has become an area of intense research. However, most traditional AIEgens suffer from undesired aggregation in aqueous media with “always on” fluorescence, or their targeting effects cannot be maintained accurately in live cells with the microenvironment changes. These drawbacks seriously impede their application in the fields of bio-imaging and antitumor therapy, which require a high signal-to-noise ratio. Herein, we propose a molecular design strategy to tune the dispersity of AIEgens in both lipophilic and hydrophilic systems to obtain the novel near-infrared (NIR, ∼737 nm) amphiphilic AIE photosensitizer (named **TPA-S-TPP**) with two positive charges as well as a triplet lifetime of 11.43 μs. The synergistic effects of lipophilicity, electrostatic interaction, and structure-anchoring enable the wider dynamic range of AIEgen **TPA-S-TPP** for mitochondrial targeting with tolerance to the changes of mitochondrial membrane potential (Δ*Ψ*_m_). Intriguingly, **TPA-S-TPP** was difficult for normal cells to be taken up, indicative of low inherent toxicity for normal cells and tissues. Deeper insight into the changes of mitochondrial membrane potential and cleaved caspase 3 levels further revealed the mechanism of tumor cell apoptosis activated by AIEgen **TPA-S-TPP** under light irradiation. With its advantages of low dark toxicity and good biocompatibility, acting as an efficient theranostic agent, **TPA-S-TPP** was successfully applied to kill cancer cells and to efficiently inhibit tumor growth in mice. This study will provide a new avenue for researchers to design more ideal amphiphilic AIE photosensitizers with NIR fluorescence.

## Abstract 2

In this contribution, based on a “step-by-step” molecular design strategy, a novel NIR amphiphilic AIEgen **TPA-S-TPP** with a triplet lifetime of 11.43 μs and surmounting the shackle of MMP was successfully fabricated for amplified tumor ablation.

## Graphical abstract

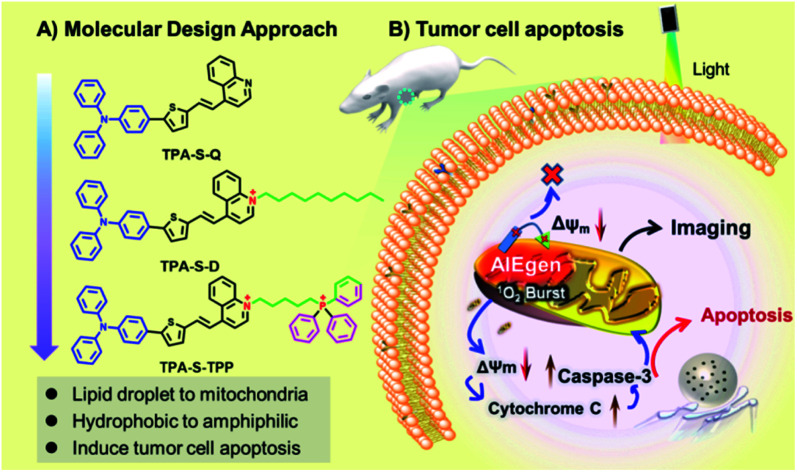

## Introduction

In relation to chemotherapy, radiotherapy and surgery,^1–5^ photodynamic therapy (PDT) is considered an ideal alternative therapeutic strategy to treat tumors,^6–11^ effectively avoiding the drawbacks of drug resistance, toxic side effects and high costs. For these reasons, PDT has been heavily investigated in recent years.^12–18^ Additionally, PDT shows high spatiotemporal selectivity and works by killing tumor cells with reactive oxygen species (ROS) or radicals produced by photosensitizers (PSs) under light irradiation.^19–22^ Increasing evidence has shown that photosensitizers located in critical positions in cancer cells can enhance the effect of phototherapy, which is mainly affected by the lifespan and activity range of ROS.^23^ As a subcellular organelle, mitochondria are the energy factories of cells and can also cause cellular suicide, regulating countless biological processes, from biosynthesis to apoptosis.^12,24–26^ Mitochondrial homeostasis plays an important role in cancer biology, including in the initiation, growth, and metastasis of cancers.^27^ Regulating the ROS and radical levels in mitochondria to activate the cell death mechanism has been regarded as a key therapeutic target for different types of cancers, and is a promising treatment for efficient tumor ablation.^12,23,28^ Actually, the ROS produced by commonly used photosensitizers targeted to the mitochondria cause a decrease in the mitochondrial membrane potential.^12^ This greatly weakens the targeting effect owing to the decrease of electrostatic interaction, a common phenomenon in mitochondrial fluorescent dye research,^29–31^ probably leading to photosensitizer leakage and further limiting its ability to effectively kill cancer cells, which ultimately weakens the therapeutic effect. In addition, the decrease in fluorescence quantum yield and ROS production caused by the aggregation-caused quenching (ACQ) of most conventional photosensitizers with large π-planar hydrophobic structures is also a therapeutic hindrance.^32^ Thus, developing a smart photosensitizer that is not affected by variations in the mitochondrial membrane potential and does not undergo ACQ is a particularly significant and challenging task at the moment.

The concept of aggregation-induced emission (AIE) was put forward by Tang *et al.* in 2001.^33^ In this phenomenon, molecules fluoresce strongly when aggregated or in a solid state, but faintly when dispersed in solution.^19,34–39^ Considering their advantages, such as high photostability, a large Stokes shift, and easy preparation, some impressive AIE luminogens (AIEgens) have been extensively explored by Tang, Liu and other groups, especially in the fields of bio-imaging, sensing, and therapy.^13,20,37,40–57^ However, undesirable aggregation of most AIEgens in aqueous environments with “always on” fluorescence is not conducive to high signal-to-noise ratio (SNR) imaging of other cellular suborganelles and targets of interest owing to their high hydrophobicity. The amphiphilic AIE strategy could achieve good dispersion with desirable “fluorescence-off” ability until the molecule reached a specific region or was activated by a specific analyst.^58,59^ Unfortunately, few studies have investigated the retention behaviour of mitochondrial targeted AIE probes after the fluctuations of mitochondrial membrane potential, to the best of our knowledge.^60^ And, the detailed mechanisms of the antitumor effects of most AIE molecules have not been well elucidated. Additionally, when the fluorescence emission reaches the biological window (650–900 nm),^61^ AIEgens can effectively avoid the interference of spontaneous fluorescence in biological tissues, improve tissue penetration, and are more conducive to biological applications in imaging-guided therapy.^62^ Thus, it is urgent to investigate amphiphilic AIE PSs with NIR fluorescence, high singlet oxygen (^1^O_2_) productivity, and excellent photostability and to systematically examine their working mechanism, effectively track their dynamic biological processes, and promote up-coming clinical biomedical application.

**Scheme 1:**
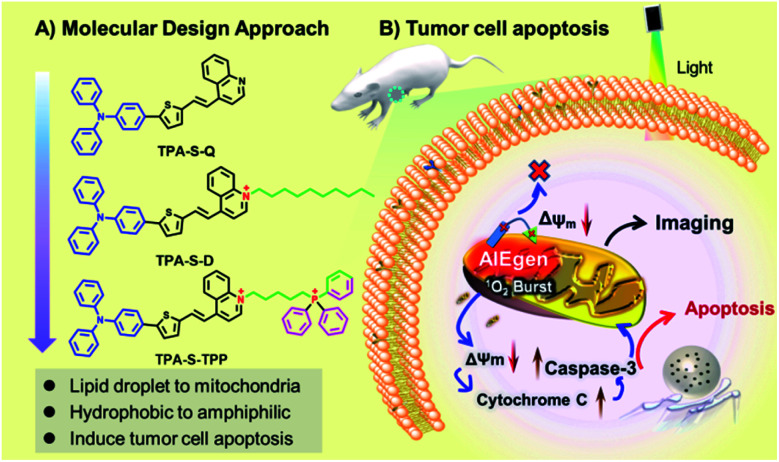
(A) Molecular design strategy for constructing the **TPA-S-Q**, **TPA-S-D**, and **TPA-S-TPP** probes. (B) Schematic illustration of AIEgen **TPA-S-TPP** anchoring to mitochondria to induce the apoptosis of tumor cells under light irradiation.

To address the bottleneck of AIE materials mentioned above, the “step-by-step” molecular design strategy was carried out to design the novel NIR amphiphilic AIEgen **TPA-S-TPP** with two positive charges for overcoming photosensitizer leakage from mitochondria. As demonstrated in Scheme 1A, triphenylamine (TPA), a classic electron-donating and hydrophobic part, was conjugated to the electron-withdrawing quinolone cationic (a hydrophilic electron acceptor) *via* a thiophene-bridged unit to obtain the amphiphilic AIEgen **TPA-S-TPP**, which has an NIR emission at 737 nm owing to the lower bandgap compared with **TPA-S-Q**. In this case, the introduced triphenylphosphine (TPP) cationic played two vital roles: realizing mitochondria targeting by virtue of its inherent accumulation character^63^ and further improving the hydrophilic dispersity compared to **TPA-S-D**. The synergistic effects of lipophilicity, electrostatic interaction, and structure-anchoring enable the wider dynamic range of AIEgen **TPA-S-TPP** for mitochondrial targeting upon the change of Δ*Ψ*_m_: (1) the lipophilic part of the structure is easily inserted into the mitochondrial membrane; (2) the electrostatic interaction between two positive charges of the AIEgen and mitochondria is stronger, compared to one positive charge of common AIE probes; (3) the two charges are connected by a flexible carbon chain that allows the AIE probe to anchor firmly to the mitochondrial membrane with tolerance to the Δ*Ψ*_m_ changes (as demonstrated in Scheme 1B). **TPA-S-TPP** has a good dispersity in both lipophilic and hydrophilic systems, essentially avoiding the “false” positive signal caused by undesirable aggregation. This performance enabled NIR amphiphilic **TPA-S-TPP** to overcome the bottleneck of traditional AIE probes suffering from “always-on” fluorescence and no target ability. Additionally, the **TPA-S-TPP** was also used to distinguish cancer cells (HeLa cells) from normal cells (L929 cells) through confocal fluorescence imaging. And, the mechanism of AIEgen **TPA-S-TPP** against cancer cells was systematically studied through toxicity tests, ROS tracing, immunofluorescent staining, and western blot analysis. Combined with its high singlet oxygen productivity, NIR amphiphilic **TPA-S-TPP** as an ideal AIE photosensitizer was applied for high signal-to-noise fluorescence-guided tumor ablation *in vivo*. The detailed synthetic routes of **TPA-S-Q**, **TPA-S-D**, and **TPA-S-TPP** are shown in Fig. S1.† Their chemical structures and intermediates were fully characterized by ^1^H-NMR, ^13^C-NMR, and high-resolution mass spectrometry (ESI-HRMS), and details are provided in the ESI Section (Fig. S2–S20†).

## Results and discussion

### Spectroscopic characteristics

**Fig. 1:**
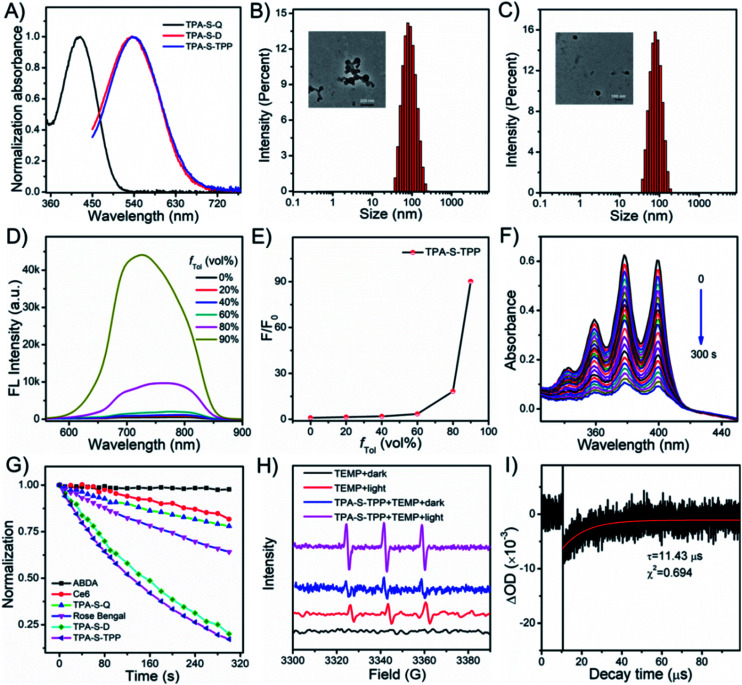
(A) Normalization absorbance of **TPA-S-Q** (10 μM), **TPA-S-D** (10 μM), and **TPA-S-TPP** (10 μM). DLS and TEM characterization of **TPA-S-Q** (10 μM, B) and **TPA-S-D** (10 μM, C). (D) Fluorescence spectra and plots of maximum fluorescence intensity changes (E) of **TPA-S-TPP** (10 μM) in the DMSO/toluene mixed solution system (toluene fraction *f*_Tol_: 0–90%). (F) Absorbance spectra changes of ABDA (50 μM) in the presence of **TPA-S-TPP** (10 μM) under white light irradiation (25 mW cm^−2^) for different times (0–300 s). (G) Normalization decomposition rates of ABDA (50 μM, absorbance at 378 nm) with Ce6 (10 μM), **TPA-S-Q** (10 μM), rose bengal (10 μM), **TPA-S-D** (10 μM), and **TPA-S-TPP** (10 μM) under white light irradiation (25 mW cm^−2^) for different times. (H) Electron spin resonance (ESR) measurement of TEMP and TEMP + **TPA-S-TPP** in the dark and light, respectively. (I) Triplet excited state lifetime of **TPA-S-TPP** (10 μM) was measured by the laser flash photolysis.

Initially, the photophysical properties of the compounds were investigated. **TPA-S-Q** displayed an absorption peak at 424 nm in DMSO (Fig. 1A). Satisfactorily, **TPA-S-D** and **TPA-S-TPP** prepared by introducing different side substituent moieties exhibited similar absorption peaks at 535 nm with a redshift of 111 nm, which were suitable for white light irradiation. As observed in Fig. S21,†**TPA-S-Q** showed bright fluorescence in organic solvents (ACN, DMSO, and EtOH) but was largely quenched in aqueous solution (DW and PBS), which was likely consistent with the typical aggregation-caused quenching (ACQ) phenomenon. This property of **TPA-S-Q** was confirmed in the commonly employed binary solvent systems of ACN/DW, DMSO/DW, and EtOH/DW with different volume fractions of water (*f*_DW_). The experimental results showed that the emission of **TPA-S-Q** dramatically decreased with an increasing fraction of DW volume, accompanied by a blueshift in the emission peaks (Fig. S22–S24†). The dynamic light scattering (DLS) and transmission electron microscopy (TEM) characterization results confirmed the nanoparticle formation of **TPA-S-Q** in aqueous solution (Fig. 1B). Additionally, in the DMSO/toluene mixture solution, the emission peak of **TPA-S-Q** also appeared blueshifted, but its fluorescence intensity did not change significantly (Fig. S25†). In contrast, the introduction of quinoline cation endowed AIEgen **TPA-S-D** with the “fluorescence-off” property in various solutions (Fig. S26†). We also studied the spectral behaviors of **TPA-S-D** in ACN/DW, DMSO/DW, and EtOH/DW (Fig. S27†). No fluorescence signals were observed upon increasing the *f*_DW_ value, which was attributed to the loose aggregation state of **TPA-S-D** in DW without restricted intramolecular rotation. And, DLS and TEM for **TPA-S-D** nanoparticles in aqueous media revealed that it had good particle size distribution (Fig. 1C). Besides, **TPA-S-D** was insoluble in low-polarity solvents such as toluene, hexane, and ether. Therefore, the AIE features of **TPA-S-D** were evaluated in the DMSO/toluene mixture solution. As shown in Fig. S28,† AIE fluorescence occurred when *f*_Tol_ in the mixed solvent exceeded 80%. Moreover, the triphenylphosphine cation intensified the amphiphilicity of **TPA-S-TPP** and further quenched its fluorescence (Fig. S29†). To verify that the NIR amphiphilic AIEgen was in an initial “fluorescence-off” state, the same method was used to evaluate the spectral behaviours of **TPA-S-TPP** in ACN/DW, DMSO/DW, and EtOH/DW (Fig. S30†). There was no fluorescence signal with the increase of *f*_DW_ value, indicating the amphiphilicity of AIEgens and their good dispersion with free intramolecular rotation. The intrinsic NIR fluorescence signals sharply and adequately increased owing to the formation of tight nanoaggregates when the *f*_Tol_ was higher than 80% (Fig. 1D), which was further confirmed by the Tyndall effect (Fig. S31†) and fluorescence confocal imaging (Fig. S32†). Additionally, the fluorescence intensity of **TPA-S-TPP** increased considerably more than that of **TPA-S-D** due to its more hydrophilic nature (Fig. 1E and S28B†).

### Singlet oxygen detection

Next, the efficiencies of the compounds to generate singlet oxygen (^1^O_2_) were investigated under white light irradiation (25 mW cm^−2^) by applying 9,10-anthracenediyl-bis(methylene)dimalonic acid (ABDA, a commercially available ^1^O_2_ indicator).^64^ As observed in Fig. 1F, with the extension of irradiation time (0–300 s), the ABDA absorption peak decreased rapidly due to the ^1^O_2_ generation (Fig. S33†), while the absorption peak of **TPA-S-TPP** remained unchanged (Fig. S34†), indicating that **TPA-S-TPP** possessed exceptional ^1^O_2_ generation ability and kept high photostability. We also tested the singlet oxygen production capacity of other compounds, including the commercial photosensitizers Ce6 and Rose Bengal (RB). The results confirmed that the constructed AIEgen **TPA-S-TPP** had excellent photodynamic effects (Fig. 1G and S35†). Additionally, electron spin resonance (ESR) was employed to verify the ^1^O_2_ generation of **TPA-S-TPP** under light irradiation by using 2,2,6,6-tetramethyl-4-piperidinol (TEMP) as an ^1^O_2_ trapper.^65^ As observed in Fig. 1H, the ESR signals of TEMP were weak under both dark and light irradiation. However, there was a remarkable ESR signal for the **TPA-S-TPP** + TEMP solution under light irradiation compared to dark conditions, which further confirmed its ability to generate ^1^O_2_. To explain the mechanism by which the AIEgen generated ^1^O_2_, laser flash photolysis was used to test the triplet-state lifetime of **TPA-S-TPP**. As seen in Fig. 1I, the lifetime of the triplet state of **TPA-S-TPP** was measured to be 11.43 μs, which indicated an energy transfer from AIEgens to other chemical substances easily (Fig. S36†), leading to an improved ^1^O_2_ generation efficiency.

### Cell imaging

**Fig. 2:**
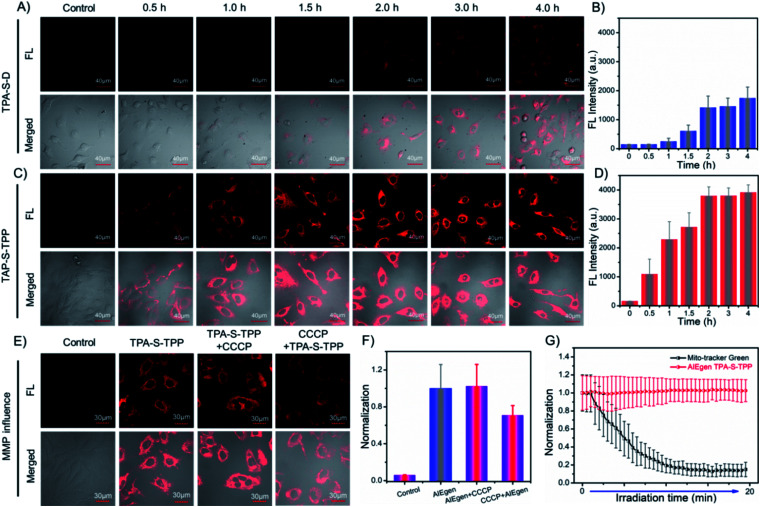
Time-dependent fluorescence images of HeLa cells incubated with **TPA-S-D** (5 μM, A) and **TPA-S-TPP** (5 μM, C) for various times. (B) and (D) Fluorescence intensities gathered from HeLa cell images (A and C). *λ*_ex_ = 559 nm and *λ*_em_ = 655–755 nm. Scale bar = 40 μm. (E) Confocal fluorescence images of HeLa cells to investigate the influence of mitochondrial membrane potential by CCCP: control group; treated with AIEgen **TPA-S-TPP**; pre-incubated with AIEgen **TPA-S-TPP** followed by adding CCCP (10 μM, 20 min); pre-incubated with CCCP (10 μM, 20 min) followed by adding AIEgen **TPA-S-TPP**. Scale bar = 30 μm. (F) Normalized fluorescence intensities based on the results of HeLa cell imaging (E). Note: the pixel intensity of the AIEgen **TPA-S-TPP** group was defined as 1.0. (G) Normalized fluorescence intensities (*F*/*F*_0_) of HeLa cells from the green channel (Mito-Tracker Green) and red channel (**TPA-S-TPP**) under continuous laser irradiation, respectively.

Encouraged by the desired behaviors in solution of the AIEgens, cell imaging was performed to explore their ability to be taken up by cancer cells. As depicted in Fig. 2C, incubation of HeLa cells for 2 h with amphiphilic **TPA-S-TPP** led to an obvious NIR fluorescence signal with a high signal-to-noise ratio (Fig. 2D), clearly indicating the good internalization of **TPA-S-TPP**. In sharp contrast, weaker fluorescence was observed in images of HeLa cells treated with **TPA-S-D** (Fig. 2A), indicative of poor penetration efficiency. In addition, semi-quantitative statistics describing the cellular uptake is displayed in Fig. 2B. A plausible explanation for the low cellular uptake of **TPA-S-D** is that long hydrophobic alkyl chains limit its ability to cross the cell membranes. In view of this, the subsequent studies mainly focused on amphiphilic AIEgen **TPA-S-TPP**. To our surprise, under the same conditions, AIEgen **TPA-S-TPP** was difficult to be taken up by L929 cells (normal cells, Fig. S37†), laying the foundation for low toxicity to normal cells and tissues during the phototherapy process. To explore the intracellular distribution of AIEgen **TPA-S-TPP** in living cells, co-localization experiments of HeLa cells incubated with **TPA-S-TPP** and commonly used commercial dyes (Hoechst 33 342, Lyso-Tracker Green, Mito-Tracker Green, and BODIPY 493/503, respectively) were subsequently carried out. As shown in Fig. S38,† the remarkable NIR fluorescence signal of **TPA-S-TPP** (Fig. S38C2†) and the green fluorescence signal of the mitochondrial-tracker (Fig. S38C1†) almost completely overlapped (Fig. S38C3†) with a higher Pearson's coefficient of 0.86 (Fig. S38C4†). There was more overlap in the mitochondria than in the nucleus (Fig. S38A1–A4,†*P* = 0.32), lysosome (Fig. S38B1–B4,†*P* = 0.45), and lipid droplets (Fig. S38D1–D4,†*P* = 0.34), owing to the synergistic effects of its lipophilicity, the electrostatic interaction, and structure-anchoring effect for selective accumulation in the mitochondria. These results clearly indicated that AIEgen **TPA-S-TPP** possessed good mitochondria targeting ability. In addition, **TPA-S-Q**-treated HeLa cells displayed bright yellow spots under a confocal microscope (Fig. S39 and S40†), which inferred that **TPA-S-Q** mainly accumulated in cytosolic lipid droplets because of its higher lipophilicity. The cell imaging results of co-localization of **TPA-S-Q** co-incubated with nile red (a commercial tracker for lipid droplets, Fig. S43†), Mito-Tracker Deep Red (Fig. S41†), and Lyso-Tracker Deep Red (Fig. S42†), respectively, further validated the above speculation. Taken together, the results of cell imaging demonstrated that our rationally designed probes could shuttle between the suborganelles of living cells, from lipid droplets (**TPA-S-Q**) to mitochondria (**TPA-S-TPP**), as a proof of concept (shown in Scheme 1).

### Effect of mitochondrial membrane potential change on AIEgens

The ^1^O_2_ produced by photosensitizers typically reduces the mitochondrial membrane potential (Δ*Ψ*_m_, MMP),^12^ which probably affects the binding degree of common photosensitizers to mitochondria owing to the decrease of electrostatic interaction, further weakening their antitumor effects. Subsequently, to prove that AIEgen **TPA-S-TPP** could be permanently located in mitochondria, carbonyl cyanide 3-chlorophenylhydrazone (CCCP), an agent that dissipates the MMP, was employed to carry out the following experiments.^31,66^ HeLa cells treated with AIEgen **TPA-S-TPP** exhibited an obvious NIR fluorescence signal, as shown in Fig. 2E. On the basis of the above experiment, CCCP was added to destroy the membrane potential of mitochondria.^31^ And, we still observed a significant fluorescence signal, similar to untreated cells, which demonstrated that the decrease of mitochondrial membrane potential had no effect on our AIE PS (Fig. 2F). Surprisingly, HeLa cells pre-incubated with CCCP followed by the addition of **TPA-S-TPP** also showed a distinct fluorescence signal (Fig. 2E and F), which exhibited the wider dynamic range of **TPA-S-TPP** for mitochondrial targeting with tolerance to the changes of Δ*Ψ*_m_. However, when the cells were incubated with CCCP first followed by adding commercial dye Mito-Tracker Red CMXRos or Rhodamine 123, compared to untreated cells, the fluorescence signal became very weak (Fig. S44†) owing to their reduced sensitivity to mitochondria. This means that the commercial mitochondrial dyes were strongly dependent on the mitochondrial membrane potential. Moreover, the commercial mitochondrial dyes did not show ideal retention when the Δ*Ψ*_m_ decreased (Fig. S44†). These results strongly confirmed that AIEgen **TPA-S-TPP** permanently stayed in the mitochondria and was not affected by fluctuations of the mitochondrial membrane potential, producing a more sustained antitumor effect in this critical location of tumor cells and enhancing the AIEgens' photodynamic effect.

### Photostability evaluation

The photostability of AIEgens is another important parameter in biomedical application. Then, we investigated the photostability of **TPA-S-TPP** in living cells under continuous laser irradiation. For the sake of contrast, commercial mitochondrial tracker dye was chosen as a control group. As observed in the time-dependent cell imaging results, after continuous light irradiation for approximately 7 min, Mito-Tracker Green was photobleached to such an extent that no appreciable fluorescence signal was detected (Fig. S45 and Video S1†). Moreover, its intrinsic ACQ property was not conducive to bio-imaging (Fig. S46†). Surprisingly, there was almost no loss of fluorescence intensity for **TPA-S-TPP** even after continuous laser irradiation for about 20 min (Fig. S45 and Video S2†). Additionally, as seen in Fig. 2G, the fluorescence intensity of Mito-Tracker Green was reduced to 15% of its original signal, while **TPA-S-TPP** remained at approximately 100%. These results clearly demonstrated the high photostability of AIEgen **TPA-S-TPP**, which reinforced the superiority of our AIEgen design.

### ROS detection and the cell apoptosis pathway

**Fig. 3:**
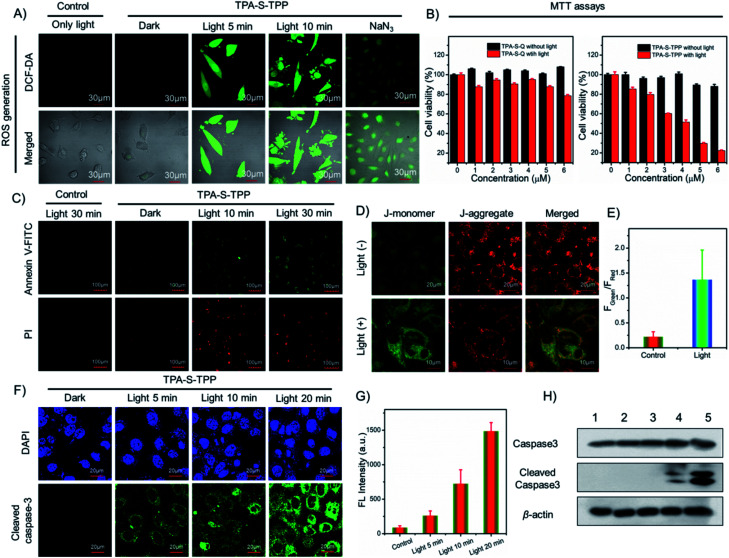
(A) ROS generation in HeLa cells using commercial dye DCF-DA after different treatments, scale bar = 30 μm. (B) MTT assays of HeLa cells treated with various concentrations (0, 1, 2, 3, 4, 5, and 6 μM) of **TPA-S-Q** (left) and **TPA-S-TPP** (right) under dark and white light irradiation, respectively. (C) Confocal fluorescence imaging of HeLa cells co-stained with annexin V-FITC and propidium iodide (PI) after different treatments: only light for 30 min; **TPA-S-TPP** + dark; **TPA-S-TPP** + light for 10 min; **TPA-S-TPP** + light for 30 min. Scale bar = 100 μm. (D) Mitochondrial membrane potential measurement of HeLa cells using commercial JC-1 dye. (E) *F*_Green_/*F*_Red_ values recorded from cells imaging using the JC-1 probe. (F) Immunofluorescence imaging analysis of HeLa cells exposed to AIEgen **TPA-S-TPP**, with (5 min; 10 min; 20 min) or without (dark group) white light irradiation. Scale bar = 20 μm. (G) Green fluorescence intensities recorded from immunofluorescence imaging (F). (H) Western blotting analysis of intracellular upregulated cleaved caspase 3 protein. Note: 1, blank + light (7 min); 2, blank + light (15 min); 3, **TPA-S-TPP** + dark; 4, **TPA-S-TPP** + light (7 min); 5, **TPA-S-TPP** + light (15 min).

Intracellular production of highly toxic ^1^O_2_ determines the efficacy of photodynamic therapy. The ^1^O_2_ production induced by AIE PS was evaluated by using commercial 2,7-dichlorofluorescein diacetate dye (DCF-DA, a fluorescent indicator of ROS generation) in live HeLa cells. Compared with the control group, there was distinct green fluorescence in HeLa cells pretreated with **TPA-S-TPP** under light irradiation (Fig. 3A), confirming the potential phototoxicity of AIE PS through the mechanism of light-mediated energy transfer (Fig. S36†). Furthermore, in the presence of sodium azide (NaN_3_, a common scavenger of ^1^O_2_), attenuated green fluorescence of HeLa cells was subsequently observed, which was ascribed to NaN_3_ efficiently inhibiting the generation of ^1^O_2_ in the cells (Fig. S47†). These experimental results demonstrated that amphiphilic **TPA-S-TPP** could cross the cell membrane and produce singlet oxygen under light-mediated conditions. Next, to quantitatively investigate the cytotoxicity of AIE PS, methylthiazolyldiphenyl-tetrazolium bromide (MTT) assays were carried out.^67^ We found that with increasing concentration of AIEgen **TPA-S-TPP**, the cell survival rate of HeLa cells remained at a very high level of approximately 90% (Fig. 3B), suggesting the low dark toxicity of **TPA-S-TPP**. However, upon white light irradiation for 30 min, a considerable decrease in cell viability was observed. These results demonstrated that **TPA-S-TPP** had the characteristics of an excellent AIE photosensitizer: negligible dark toxicity and high phototoxicity. Besides, the PDT effects of **TPA-S-Q** and **TPA-S-D** for killing cancer cells were also evaluated. As illustrated in Fig. 3B and S48,† the HeLa cells suffered only slight damage, and the viability was maintained at 78.49% and 94.27%, respectively, under photo-irradiation for 30 min. Consequently, we concluded that the designed AIEgen **TPA-S-TPP** had an ideal photodynamic effect at the cellular level.

When cell apoptosis occurs, phosphatidylserine is everted onto the surface of the cell membrane and can be detected by annexin V-FITC with high specificity and affinity. Propidium iodide (PI) is used to mark cellular DNA in necrotic cells where the cell membrane has been completely damaged. Therefore, the annexin V-FITC/PI apoptosis detection kit was employed to assess the occurrence of cell apoptosis during the PDT process. In comparison to the control group, HeLa cells pretreated with **TPA-S-TPP** exhibited no obvious fluorescence signal under dark conditions, but showed a strong green fluorescence signal from the annexin V-FITC channel and a red fluorescence signal from the PI channel (Fig. 3C) under white light irradiation, suggesting the occurrence of cell apoptosis. Furthermore, the complete destruction of the cell membrane was easily recognized by the morphology in the confocal bright field images (Fig. S49†).

To further elucidate the mechanism of AIEgen **TPA-S-TPP** inducing apoptosis of cancer cells, the JC-1 probe was first employed as an indicator for monitoring the changes of MMP.^68^ As depicted in Fig. 3D, upon photoirradiation, the green fluorescence of HeLa cells incubated with AIEgen **TPA-S-TPP** became stronger, while the red fluorescence weakened, resulting in a 6.14-fold increase (Fig. 3E) in the fluorescence intensity ratio of green-to-red compared with the “**TPA-S-TPP** + dark” group. This indicated that MMP had decreased. There is ample evidence that mitochondrial damage promotes the release of cytochrome c, further initiating the apoptotic cascade and activating caspase 3. Therefore, caspase 3 activation was assessed using immunofluorescence imaging analysis and the cleaved caspase 3 antibody. We observed that the green fluorescence signal in the HeLa cells incubated with AIEgen **TPA-S-TPP** was stronger with the extension of light irradiation compared to the control group (Fig. 3F). The details of their average fluorescence intensities are shown in Fig. 3G and were consistent with the cell imaging results.

Additionally, western blotting analysis showed an upregulated intracellular level of cleaved caspase 3 protein in HeLa cells treated with AIEgen “**TPA-S-TPP** + light” (Fig. 3H), as expected.^69^ The above experiments confirmed that AIEgen **TPA-S-TPP** could act as an excellent photosensitizer to reduce the mitochondrial membrane potential owing to ^1^O_2_ production under light radiation. This promotes the release of cytochrome c and subsequently activates caspase 3, finally leading to cell apoptosis. This mitochondria-mediated pathway of apoptosis was consistent with the description in Fig. S50.†

### Live/dead cell staining

**Fig. 4:**
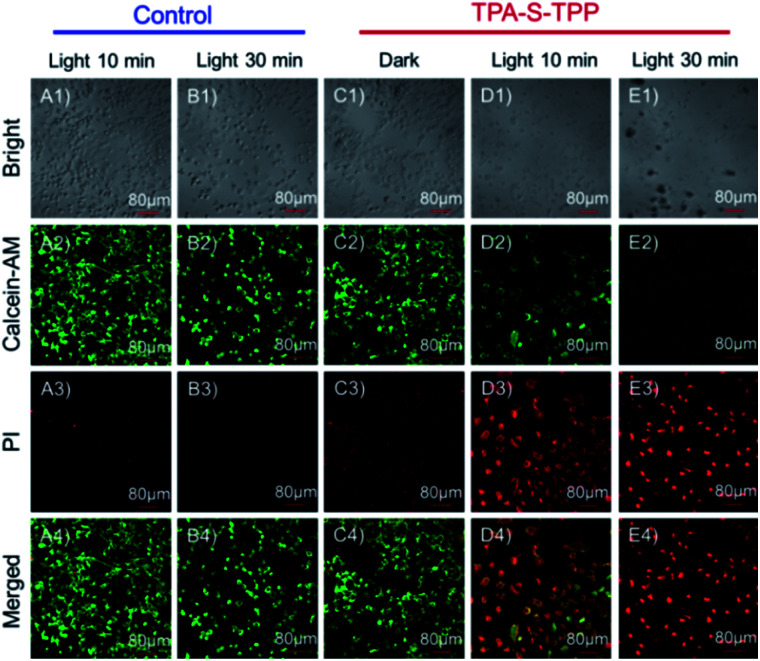
Confocal fluorescence imaging of HeLa cells co-stained with calcein-AM and propidium iodide (PI) after different treatments: (A), only light for 10 min; (B), only light for 30 min; (C), **TPA-S-TPP** + dark; (D), **TPA-S-TPP** + light for 10 min; (E), **TPA-S-TPP** + light for 30 min. Scale bar = 80 μm.

The AIEgen **TPA-S-TPP**-induced photodynamic effect was further tested by calcein-AM and propidium iodide (PI) staining.^70^ As expected, the HeLa cells in both the control group and the **TPA-S-TPP** dark group displayed strong green fluorescence (Fig. 4A2, B2 and C2), indicating that there was no damage to the cells. In contrast, the majority of the cancer cells treated with AIEgen **TPA-S-TPP** were killed under photoirradiation. PI could cross their severely damaged cell membranes and enter the nuclei, resulting in obvious red fluorescence (Fig. 4D3 and E3), whereas the green fluorescence dramatically decreased (Fig. 4D2 and E2). These results were consistent with the above experimental phenomena, clearly verifying that AIEgen **TPA-S-TPP** could induce cell apoptosis by the generation of ^1^O_2_.

### *In vivo* application

**Fig. 5:**
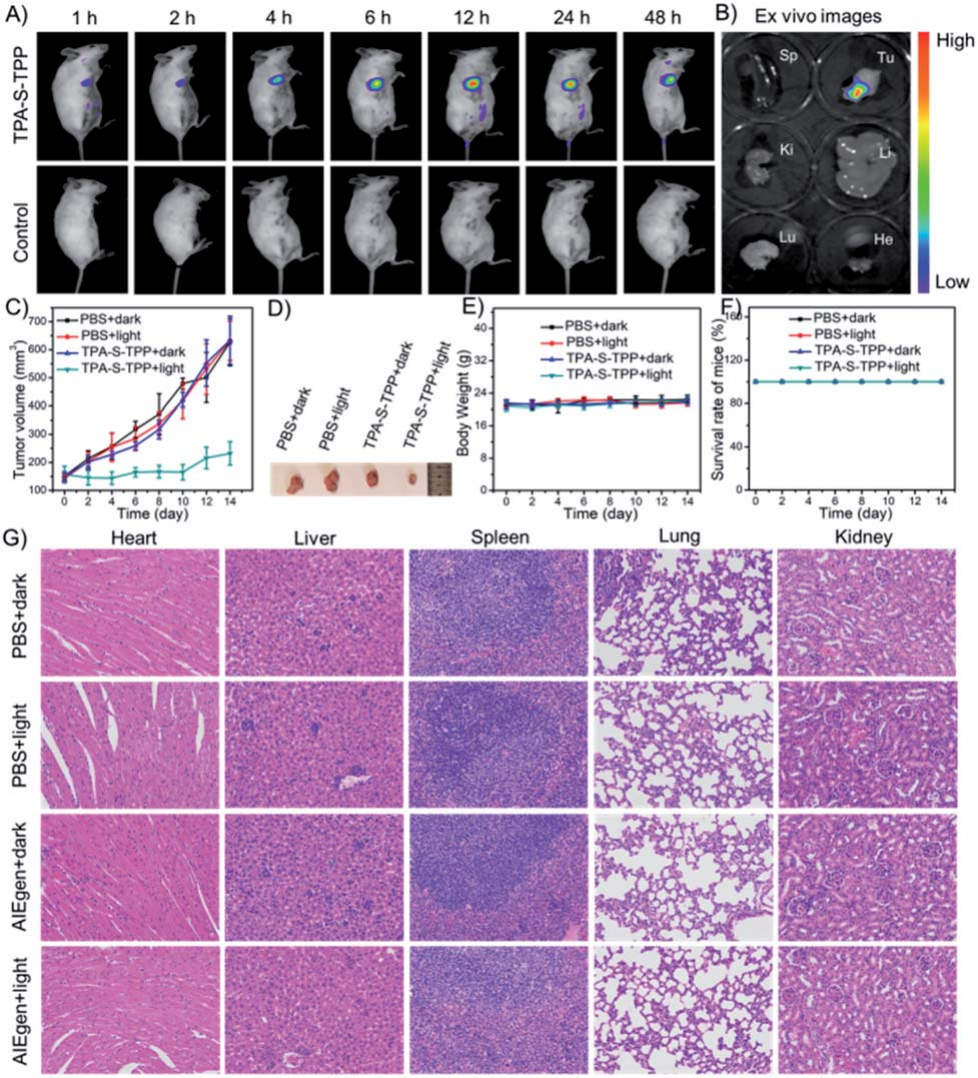
(A) Time-dependent *in vivo* images of 4T1 tumor-bearing BALB/c mice *in situ* injected with AIEgen **TPA-S-TPP** and PBS (control group). (B) *Ex vivo* images of the main organs (spleen: Sp; tumor: Tu; kidney: Ki; liver: Li; lung: Lu; heart: He). (C) Tumor volume changes of 4T1 tumor-bearing BALB/c mice during the different treatments (*n* = 5). (D) Representative tumor photos of tumor-bearing mice after treatment for 14 days. (E) Body weight changes of 4T1 tumor-bearing BALB/c mice during the different treatments. (F) Survival rates of tumor-bearing mice in different groups. (G) Hematoxylin and eosin (H&E) staining of the main organ slices (heart, liver, spleen, lung, and kidney) from different groups after treatment for 14 days.

Inspired by the promising results of spectral behavior, ^1^O_2_ production, and cell imaging, the feasibility of AIEgen **TPA-S-TPP** was explored in the 4T1-tumor BALB/c mouse model. After *in situ* injection of **TPA-S-TPP**, the *in vivo* imaging was monitored using the NightOWL II LB983 small animal imaging system. As seen in Fig. 5A, the NIR fluorescence signal in the tumor site increased gradually compared to the control group, reaching a peak after 12 h. An obvious fluorescence signal was still observed after 48 h, which further confirmed the potential phototherapy application of AIEgen **TPA-S-TPP** to inhibit tumor growth. Similar experimental results were also verified by *ex vitro* organ imaging (Fig. 5B). The PDT effect of our rationally designed AIE PS on tumors in 4T1-bearing BALB/c mice was then evaluated. The tumor-bearing mice were randomly divided into 4 groups (*n* = 5): 1, “PBS + dark”; 2, “PBS + light”; 3, “**TPA-S-TPP** + dark”; and 4, “**TPA-S-TPP** + light”. As shown in Fig. 5C, the tumor growth in the first three groups proceeded normally after treatment for 14 days, indicating that light exposure or AIEgen **TPA-S-TPP** under dark conditions had a negligible effect on inhibiting tumor growth. By contrast, upon treatment with the AIEgen and photoirradiation, tumor growth was significantly inhibited (Fig. 5C), indicating a high therapeutic effect of **TPA-S-TPP**. Representative tumor photos of mice after treatment for 14 days are shown in Fig. 5D, and the results were consistent with the above photodynamic therapy trends.

### Biosafety assessment

An assessment of the biosafety of AIE materials is necessary before they can be clinically applied. There was no significant difference in the body weights of BALB/c mice between the “**TPA-S-TPP** + light” group and the other groups (Fig. 5E), and no mice deaths occurred during the course of treatment (Fig. 5F). Additionally, four groups of mice were euthanized at the end of the treatment cycle, and their main organs (heart, liver, spleen, lung, and kidney) were made into paraffin sections using a standard method and stained with hematoxylin and eosin (H&E). As observed in Fig. 5G, no noticeable inflammation or cell necrosis lesions were found in any of the major organs. Taken together, these results confirmed that **TPA-S-TPP** could serve as an efficient AIE PS with high biocompatibility for tumor ablation *in vivo*.

## Conclusions

To address the “always on” fluorescence that hinders traditional AIEgens, we fabricated a novel NIR amphiphilic AIEgen **TPA-S-TPP** with two positive charges based on a “step-by-step” molecular design strategy. This AIEgen **TPA-S-TPP** exhibited a “fluorescence-off” state before tightly locating to the mitochondria, effectively avoiding a false positive signal. Compared with **TPA-S-Q**, **TPA-S-TPP** could better escape from the lipid droplet region to the mitochondria. Compared with **TPA-S-D**, **TPA-S-TPP** more easily entered cancer cells and demonstrated good biocompatibility. The decrease in mitochondrial membrane potential had almost no effect on the AIEgen **TPA-S-TPP** by virtue of the synergistic effects of lipophilicity, the electrostatic interaction, and structure-anchoring, which was an improvement over the tested commercial Mito-tracker dyes. Additionally, AIEgen **TPA-S-TPP** could be employed to distinguish cancer cells from normal cells through confocal fluorescence imaging. These attributes, along with high singlet oxygen generation ability better than commonly used commercial dyes (Ce6 and Rose Bengal) and excellent photostability, enabled the amphiphilic **TPA-S-TPP** to efficiently kill cancer cells through enhanced PDT. The apoptosis pathway of cancer cells was clarified in detail by fluorescence imaging, immunofluorescence imaging, and western blot experiments. Tumor-bearing mouse experiments showed that **TPA-S-TPP** could also effectively inhibit tumor growth by PDT. Furthermore, H&E staining of the major organs demonstrated its safety *in vivo*. In brief, the excellent performances make AIEgen **TPA-S-TPP** an efficient agent for tumor ablation and provide guidance for developing additional NIR amphiphilic AIE PSs with high signal-to-noise ratios.

## Ethical statement

This study was conducted in accordance with the Guide for the Care and Use of Laboratory Animals. The animal protocol was approved by the local research ethics review board of the Animal Ethics Committee of Dalian University of Technology (certificate number/Ethics approval no. 2018-043).

## Author contributions

H. Li and J. Yoon conceived the ideas and planned the research. H. Li designed the synthetic route and conducted the synthesis work. H. Li, Y. Lu, and S. Long carried out characterization and the *in vitro* experiments. H. Li, G. Kim, J. Han, J. Chung, Y. Lu, and H. Kim performed cell experiments. Y. Lu and Q. Yao performed the mice experiments and H&E tissue staining. X. Wu analyzed data and contributed to the scientific discussions on the manuscript. H. Li wrote the manuscript. X. Peng and J. Yoon revised the manuscript and supervised the research.

## Conflicts of interest

The authors declare no competing financial interests.

## Supplementary Material

SC-012-D1SC02227J-s001

SC-012-D1SC02227J-s002

SC-012-D1SC02227J-s003
