# Supplementary material for: Activation of apoptosis by rationally constructing NIR amphiphilic AIEgens: surmounting the shackle of mitochondrial membrane potential for amplified tumor ablation
Source: Chem Sci. 2021 Jul 6;12(31):10522–31. doi: 10.1039/d1sc02227j (PMC8356816; doi:10.1039/d1sc02227j)
Supplement: SC-012-D1SC02227J-s001 [file SC-012-D1SC02227J-s001.pdf]

Electronic Supplementary Material (ESI) for Chemical Science.
This journal is © The Royal Society of Chemistry 2021

Activation of Apoptosis by Rationally Constructing NIR Amphiphilic AIEgens: Surmounting the Shackle of Mitochondrial Membrane Potential for Amplified Tumor Ablation

Haidong Li,[a,d] Yang Lu,[b,d] Jeewon Chung,[a] Jingjing Han,[a] Heejeong Kim,[a] Qichao Yao,[b,c] Gyoungmi Kim,[a] Xiaofeng Wu,[a] Saran Long,[b] Xiaojun Peng[*,b], and Juyoung Yoon[*,a]

[a] Department of Chemistry and Nanoscience, Ewha Womans University, Seoul 03760, Korea

[b] State Key Laboratory of Fine Chemicals, Dalian University of Technology, 2 Linggong Road, Hi-tech Zone, Dalian 116024, China

[c] Ningbo Institute of Dalian University of Technology, Dalian University of Technology, 26 Yucai Road, Jiangbei District, Ningbo 315016, China

[d] These authors contributed equally to this work.

* Address correspondence to pengxj@dlut.edu.cn and jyoon@ewha.ac.kr

EXPERIMENTAL DETAILS

All the reagents used were acquired from commercial suppliers and were used without further purification unless otherwise noted. Solvents used were purified via standard methods. ^{1}H, ^{13}C, and ^{31}P-NMR spectra were measured on a Bruker 300 MHz spectrometer. Chemical shifts (δ) were reported as ppm (using TMS as the internal standard). Fluorescence spectra were measured on a FS-2 fluorescence spectrophotometer (Scinco). Excitation and emission slit widths were modified to adjust the fluorescence intensity to a suitable range. Absorption spectra were measured on Thermo Scientific Evolution 201 UV-Vis spectrophotometer. Mass spectrometric data were achieved with SYNAPT G2 (Waters, U.K.). Dynamic light scattering (DLS) was measured using a Nano-ZS (Malvern). EPR analysis was conducted on EMX-plus equipment (Bruker). Transmission Electron Microscope (TEM) images were obtained using JEM-2100F (JEOL) using a 100 kV. Cell imaging tests were carried out on Olympus FV 1200 confocal microscopy (Olympus, Japan). Distilled de-ionized water (DW) was prepared by laboratory ultrafiltration.

Singlet oxygen ($^{1}O_2$) generation

9,10-Anthracenediyl-bis(methylene)dimalonic acid (ABDA, 50 μM) was used to detect singlet oxygen generation of Ce6 (10 μM), Rose Bengal (10 μM), TPA-S-Q (10 μM), TPA-S-D (10 μM), and TPA-S-TPP (10 μM) upon light irradiation. Brief, The sample mixtures (ABDA + probes in the solution) were then irradiated under white light irradiation (25 mW/cm^{2}) at intervals of 10 s until 300 s. The absorbance spectra of ABDA (50 μM) were recorded by the Thermo Scientific Evolution 201 UV-Vis spectrophotometer. Additionally, the absorption of ABDA at 378 nm was measured at various irradiation times (0-300 s) to gather the decay rate of photosensitizing process.

Laser flash photolysis

Nanosecond time-resolved transient absorption spectra were recorded on a LP980 laser flash photolysis spectrometer (Edinburgh Instruments Ltd.) in combination with a Nd:YAG laser (Surelite I-10, Continuum Electro-Optics, Inc.). Sample in nitrogen atmosphere was excited by a 355 nm laser pulse (1 Hz, 100 mJ per pulse, fwhm $\approx$ 7 ns) at room temperature. Concentration of TPA-S-TPP was 10 μM with absorbance lower than 0.5 OD at 355 nm in 1 cm path length quartz cuvettes.

Cell incubation

Human cervical carcinoma cells (HeLa cells) were obtained from Korean Cell Line Bank (Seoul, Korea). 4T1 breast cancer cells were purchased from Institute of Basic Medical Sciences (IBMS) of the Chinese Academy of Medical Sciences (China). Cells were cultured in medium supplemented with 10% fetal bovine serum (Invitrogen) according to the guideline of ATCC. The cells were seeded in confocal culture dishes and then incubated for 24 h at 37 ºC under a humidified atmosphere containing 5% CO_2.

Cell imaging

HeLa cells were seeded in the culture dishes at approximately concentration of 1 × 10^5 cells/mL and allowed to culture for 24 h at 37 ºC in a 5% CO_2 humidified incubator. Then, the culture medium was remove and added Hank's Balanced Salt Solution (HBSS) containing the TPA-S-D and TPA-S-TPP (5 μM) for 0, 0.5 h, 1.0 h, 1.5 h, 2.0 h, 3.0 h, and 4.0 h at 37 ºC under a humidified atmosphere containing 5% CO_2, followed by washing thrice with DPBS. Under the confocal fluorescence microscope with a 60 × objective lens, AIEgens were excited at 559 nm (FV 1200, Olympus) and the emission was collected at 655-755 nm.

Co-localization experiments

HeLa cells were seeded in the culture dishes at approximately concentration of 1 × 10^5 cells/mL and allowed to culture for 24 h at 37 ºC in a 5% CO_2 humidified incubator. The culture medium was remove and added Hank's Balanced Salt Solution (HBSS) containing the TPA-S-TPP (5 μM) for 1.5 h at 37 ºC under a humidified atmosphere containing 5% CO_2. Then, commercial Hoechst 33342, Lyso-tracker green, and Mito-tracker green were added into the cells under the direction of the instructions, respectively. After the incubation of another 30 min, HeLa cells were washed twice with DPBS and imaged by FV 1200 confocal microscopy.

Mitochondria-locating of AIEgen affected by CCCP

HeLa cells were seeded in the culture dishes at approximately concentration of 1 × 10^5 cells/mL and allowed to culture for 24 h at 37 ºC in a 5% CO_2 humidified incubator. The culture medium was remove and added HBSS containing the TPA-S-TPP for 2 h. and then added CCCP (10 μM) for another 20 min incubation. Cells were washed twice with DPBS and imaged by FV 1200 confocal microscopy. Similar to the above method, the order of addition should be reversed (CCCP then probes) and then the cells imaged by confocal fluorescence microscopy.

HeLa cells were treated with Mito-tracker Red CMXRos (200 nM) for 20 min, and then added CCCP (10 μM) for

another 20 min incubation. Cells were washed twice with PBS and imaged by FV 1200 confocal microscopy. Similar to the above method, the order of addition should be reversed (CCCP then probes) and then the cells imaged by confocal fluorescence microscopy.

Similarly, HeLa cells were treated with Rhodamine 123 (2 μM) for 20 min, and then added CCCP (10 μM) for another 20 min incubation. Cells were washed twice with PBS and imaged by FV 1200 confocal microscopy. Similar to the above method, the order of addition should be reversed (CCCP then probes) and then the cells imaged by confocal fluorescence microscopy

Intracellular singlet oxygen detection

Singlet oxygen ($^1O_2$) generation inside cells under white light irradiation was measured using DCF-DA (2, 7-dichlorofluorescein diacetate) Detection Kit. HeLa cells were cultured in the confocal culture dishes at 37 ºC. The culture medium was remove and added Hank's Balanced Salt Solution (HBSS) containing the TPA-S-TPP (5 μM) for 1 h at 37 ºC under a humidified atmosphere containing 5% CO_2. Then, DCF-DA (10 μM) was added into the cells. After the incubation of 20 min, HeLa cells were washed twice with DPBS and then exposed to white light irradiation (60 mW/cm^2) for various time (0, 5 min, and 10 min). Additionally, the NaN_3 (50 μM) was also added into cells before photo irradiation for the consumption of ROS generation. In control group, cells only were treated with DCF-DA (10 μM). The cell images were obtained using the FV 1200 confocal microscopy with the excitation of 473 nm and the emission of 490-540 nm.

MTT assays

Measurement of cell viability was tested by reducing of 3-(4, 5)-dimethylthiahiazo (-2-yl)-3, 5-diphenytetrazoliumromide (MTT) to formazan crystals using mitochondrial dehydrogenases. HeLa cells were seeded in 96-well microplates (Nunc, Denmark) at a density of 1×10^5 cells/mL in 100 μL medium containing 10% FBS. After 24 h of cell attachment, the cells were then cultured in free medium (without FBS) containing various concentrations of probes for 2 h incubation at 37 ^oC. Then, the free medium was changed to medium supplemented with 10% FBS and irradiated with or without white light (100 mW/cm^2) for 30 min. After 24 h incubation, MTT (5 mg/mL) was added to each well and the plates were incubated at 37 ºC for another 4 h in a 5% CO_2 humidified incubator. The medium was then carefully removed, and the purple crystals were lysed in 100 μL DMSO for 15~20 min. Optical density of solutions was determined on a microplate reader at 650 nm. Cell viability was expressed as a percent of the control

culture value, and it was calculated using the following equation,

$$\text{Cells viability (\%)} = (OD_{dye} - OD_{blank}) / (OD_{control} - OD_{blank}) \times 100$$

Annexin V-FITC/propidium iodide (PI) apoptosis detection

HeLa cells were cultured in the confocal culture dishes at 37 ºC under a humidified atmosphere containing 5% CO_2. Then, cells were treated with following different treatments: group 1, irradiated with white light for 30 min (Control); group 2, incubated with TPA-S-TPP (5 μM) for 2 h at 37 ºC (Dark); group 3, incubated with TPA-S-TPP (5 μM) for 2 h at 37 ºC and irradiated with white light for 10 min (TPA-S-TPP + light 10 min); group 4, incubated with TPA-S-TPP (5 μM) for 2 h at 37 ºC and irradiated with white light for 30 min (TPA-S-TPP + light 30 min). After different treatments, HeLa cells were stained with Annexin V-FITC/propidium iodide (PI) Apoptosis Detection Kit according to the manufacture instruction and imaged by FV 1200 confocal microscopy.

Mitochondria membrane potential (MMP) analysis

HeLa cells were cultured in the confocal culture dishes at 37 ºC under a humidified atmosphere containing 5% CO_2. HeLa cells were then washed by DPBS and incubated with HBSS containing TPA-S-TPP (5 μM) for 2 h. The cells were washed again with DPBS, added fresh cell culture medium, and then irradiated with white light for 20 min. After 30 min incubation under cell incubator, HeLa cells were treated with fresh cell culture medium containing fluorescent probe JC-1 (2 μg/mL) at 37 °C according to the manufacture instruction. Finally, the cells were washed with DPBS three times and imaged by the FV 1200 confocal microscopy.

Cleaved caspase 3 immunofluorescence

HeLa cells were cultured on slide for 24 h and treated with the TPA-S-TPP for 2 h and irradiated with light (0, 5 min, 10 min, and 20 min) and washed with PBS and then fixed with 4% paraformaldehyde for 15 min, followed by permeabilization with PBS containing 0.1% Triton X-100 for 15 min at room temperature. The cells were incubated with blocking buffer (10% normal goat serum in PBS) for 1 h and then incubated with primary antibodies (Cell Signaling Tech.) in 1.5% normal goat serum for overnight at 4 ºC. To remove excess primary antibodies, cells were washed with PBS three times at 5 min intervals. Secondary antibodies conjugated with Alexa Fluor 488 (Cell Signaling Tech.) were added to the cells, which were incubated in the dark for 1 h at room temperature. The HeLa cells were

then washed with PBS three times at 5 min intervals and mounted using UltraCruz Mounting Medium with DAPI (Santa Cruz Biotech.) Fluorescence images were detected using a confocal microscopy (FV1200, Olympus) with DAPI (ex. 405 nm/em. 430-455 nm) and Alexa Fluor 488 (ex. 473 nm/em. 490-590 nm).

Western blot experiment

HeLa cells were cultured on 6 well-plate for 24 h and treated by different treatments, as following: group 1, blank + light (7 min); group 2, blank + light (15 min); group 3, TPA-S-TPP (3 µM) + dark; group 4, TPA-S-TPP (3 µM) + light (7 min); group 5, TPA-S-TPP (3 µM) + light (15 min). And then rinsed with PBS and lysed with RIPA buffer (50 mM Tris-HCl, 150 mM NaCl, 1% Triton X-100, 1% sodium deoxycholate, 0.1% SDS, 1 mM EDTA, pH 7.4), 30 min in ice and centrifuged 13,000 rpm, 10 min, 4 ºC. Supernatants were collected and protein concentrations were quantified using the BCA protein assay kit (Thermo Scientific). Equivalent amounts of protein (35 µg) were separated by SDS-PAGE and transferred to PVDF (polyvinylidene difluoride) membranes. The membranes were blocked for 1 h in blocking buffer (5% nonfat dry milk in TTBS (0.1% Tween 20-TBS)) and washed with TTBS and replaced by the primary antibody diluted in blocking buffer and incubated overnight at 4 ºC. The membranes were then washed with TTBS three times and incubated with secondary antibody for 2 h and detected by Pierce ECL Western blotting substrate (Thermo Scientific). Primary antibody, caspase-3, *β*-actin and secondary antibody anti-rabbit IgG HRP-linked antibody, anti-mouse IgG HRP-linked antibody were purchased from Cell Signalling Tech.

Dead/Live cell co-staining

HeLa cells were cultured in the confocal culture dishes at 37 ºC under a humidified atmosphere containing 5% CO_2. Then, cells were treated by different treatments, as following: group 1, irradiated with white light for 10 min (Control + light 10 min); group 2, irradiated with white light for 30 min (Control + light 30 min); group 3, incubated with TPA-S-TPP (5 µM) for 2 h at 37 ºC (TPA-S-TPP + Dark); group 4, incubated with TPA-S-TPP (5 µM) for 2 h at 37 ºC and irradiated with white light for 10 min (TPA-S-TPP + light 10 min); group 5, incubated with TPA-S-TPP (5 µM) for 2 h at 37 ºC and irradiated with white light for 30 min (TPA-S-TPP + light 30 min). After treatments, HeLa cells were stained with Calcein AM and Propidium Iodide Detection Kit and imaged by FV 1200 confocal microscopy according to the manufacture instruction.

Care and use of animals

This study was conducted in accordance with the Guide for the Care and Use of Laboratory Animals. The animal protocol was approved by the local research ethics review board of the Animal Ethics Committee of Dalian University of Technology (certificate number/Ethics approval no. 2018-043). Female BALB/c mice, 6-8 weeks of age, were purchased from SPF experimental Animal Centre of Dalian Medical University. Tumor models were established by transplanting 4T1 cells (1×10^6 cells per mouse) into the selected armpit positions.

In vivo imaging and phototherapy evaluation

After some day's inoculation, tumors grew to about 150 mm^3 in volume before used for in vivo imaging and phototherapy. The xenograft tumor mice were given with AIEgen TPA-S-TPP (200 μM, 100 μL) through in situ injection within the period of mice anaesthesia. After that, in vivo images of 4T1 tumor-bearing Balb/c mice at various times (1 h, 2 h, 4 h, 6 h, 12 h, 24 h, and 48 h) were gathered through small-animal imaging equipment (NightOWL II LB983) with a 550 nm excitation laser and a 700 nm emission filter. In the part of PDT therapy, 4T1 tumor-bearing mice were randomly divided into four groups (n = 5 per group): 1, "PBS + dark"; 2, "PBS + light"; 3, "TPA-S-TPP + dark"; and "TPA-S-TPP + light". After intratumor injection of TPA-S-TPP (200 μM, 100 μL), the tumor region was exposed to 550 nm Xe lamp (200 mW/cm^2) for 30 min. Then, the tumors volume of all mice was measured every two days using a caliper for 14 days after different treatments. The tumor volumes were measured with a caliper using the following formula,

$$V\ (mm^3) = a\ (mm) \times a\ (mm) \times b\ (mm)/2$$

V represents the tumor volume; a represents the width of tumor on the mice; b represents the length of tumor on the mice. Meanwhile, the body weight of mice was also recorded using analytical balance.

H&E staining of tissue slices

After the treatment of 14 days, the mice were euthanized, and main organs (heart, liver, spleen, lung, and kidneys) were obtained for histological analysis through the standard H&E staining.

Synthesis section

Synthesis of compound 3. Compound 1 (1.94 g, 6 mM), compound 2 (1.12 g, 7.2 mM), K_2CO_3 (2.48 g, 18 mM), and tetrakis(triphenylphosphine)palladium (346 mg, 0.3 mM) were added to the MeOH/Toluene (v/v = 1:1, 100 mL)

mixture solution under N_2 atmosphere. The above mixture was stirred under reflux condition for 24 h and cooled down to room temperature. The solvent was evaporated under reduced pressure, and purified product was obtained by silica gel column chromatography, giving yellow solid (1.15 g, yield 54%). ^1H NMR (300 MHz, $CDCl_3$): δ = 9.85 (s, 1H), 7.71 (d, *J* = 4.0 Hz, 1H), 7.57-7.47 (m, 2H), 7.35-7.23 (m, 5H), 7.18–7.00 (m, 8H). ^{13}C NMR (75 MHz, $CDCl_3$): δ = 182.64, 154.61, 149.17, 146.99, 141.34, 137.78, 129.52, 127.28, 126.15, 125.20, 123.91, 122.90, 122.39. ESI-MS: m/z calcd for $C_{23}H_{17}NOS^+$ $[M]^+$ 355.1031, found 355.1026; m/z calc'd for $C_{23}H_{17}NONaS^+$ $[M+Na]^+$ 378.0923, found 378.0923.

Synthesis of compound 5. Compound 4 (1.43 g, 10 mM) and 1-Iododecane (2.68 g, 10 mM) were added to anhydrous CH_3CN (20 mL) under N_2 protection. The mixture was stirred at 85 °C for 12 h and cooled down to room temperature. The solvent was evaporated under reduced pressure, and purified product was obtained by silica gel column chromatography, giving sticky liquid (2.75 g, yield 67%). ^1H NMR (300 MHz, DMSO-d_6): δ = 9.46 (d, J = 6.1 Hz, 1H), 8.66-8.52 (m, 2H), 8.33-8.21 (m, 1H), 8.07 (ddd, *J* = 8.0, 6.5, 0.7 Hz, 2H), 5.02 (t, *J* = 7.5 Hz, 2H), 3.02 (s, 3H), 2.08-1.80 (m, 2H), 1.55-1.04 (m, 14H), 0.84 (t, *J* = 6.7 Hz, 3H). ^{13}C NMR (75 MHz, DMSO-d_6): δ = 159.00, 148.83, 137.19, 135.59, 130.07, 129.45, 127.66, 123.14, 119.87, 57.42, 31.73, 29.93, 29.32, 29.30, 29.11, 28.95, 26.21, 22.55, 20.24, 14.43. ESI-MS: m/z calc'd for $C_{20}H_{30}N^+$ $[M-I]^+$ 284.2373, found 284.2378.

Synthesis of TPA-S-D. Compound 3 (106.5 mg, 0.3 mM), compound 5 (123 mg, 0.3 mM), and piperidine (1~3 drop) were dissolved in anhydrous 20 mL EtOH under N_2 atmosphere. The mixture was stirred at 85 °C for overnight and cooled down to room temperature. The solvent was evaporated under reduced pressure. Following, the crude product was purified through silica gel column chromatography to obtain 61 mg TPA-S-D (Yield 27%). ^1H NMR (300 MHz, CD_2Cl_2): δ = 9.73 (d, *J* = 6.1 Hz, 1H), 8.62 (d, *J* = 8.4 Hz, 1H), 8.44 (d, *J* = 6.1 Hz, 1H), 8.34-8.12 (m, 3H), 8.03-7.92 (m, 1H), 7.69-7.51 (m, 4H), 7.39-7.30 (m, 5H), 7.21-7.13 (m, 5H), 7.13-7.06 (m, 3H), 5.03 (t, *J* = 7.5 Hz, 2H), 2.16-2.03 (m, 2H), 1.59-1.49 (m, 2H), 1.32 (m, 12H), 0.90 (t, *J* = 5.6 Hz, 3H). ^{13}C NMR (75 MHz, CD_2Cl_2): δ = 152.69, 150.15, 148.82, 147.01, 139.05, 138.01, 136.93, 135.59, 135.07, 129.47, 129.30, 129.07, 126.88, 126.75, 126.32, 126.15, 125.19, 123.89, 123.74, 123.24, 122.30, 118.36, 116.21, 116.09, 57.34, 31.85, 29.86, 29.47, 29.40, 29.25, 29.13, 26.54, 22.66, 13.88. ESI-MS: m/z calc'd for $C_{43}H_{45}N_2S^+$ $[M-I]^+$ 621.3298, found 621.3303.

Synthesis of TPA-S-Q. A solution of compound 3 (710 mg, 2 mM), compound 4 (572 mg, 4 mM), and benzoyl chloride (394 mg, 2 mM) in 5 mL anhydrous DMF was stirred at 60 °C for 8 h under N_2 protection. Then the mixture was

quenched by water and extracted by DCM, and dried with anhydrous Na_2SO_4. After removal of the solvent under reduced pressure by evaporation, the crude product was purified by silica column chromatography, obtaining 163 mg TPA-S-Q (Yeild 17%). ^{1}H NMR (300 MHz, $CDCl_3$): δ = 8.90 (d, J = 4.7 Hz, 1H), 8.28-8.14 (m, 2H), 7.74 (ddd, *J* = 8.3, 6.9, 1.3 Hz, 1H), 7.59 (ddd, *J* = 11.4, 7.4, 3.2 Hz, 3H), 7.53-7.39 (m, 3H), 7.34-7.23 (m, 4H), 7.16 (m, 6H), 7.12-7.03 (m, 4H). ^{13}C NMR (75 MHz, $CDCl_3$): δ = 149.81, 148.41, 147.86, 147.35, 145.17, 142.79, 142.43, 140.35, 129.97, 129.85, 129.75, 129.54, 129.44, 128.25, 128.19, 127.64, 126.61, 126.19, 124.79, 123.43, 123.29, 122.85, 120.99, 116.36. ESI-MS: m/z calc'd for $C_{33}H_{25}N_2S^+$ $[M+H]^+$ 481.1733, found 481.1737.

Synthesis of compound 8. Compound 6 (2.28 g, 10 mM) and compound 7 (2.62 g, 10 mM) were dissolved in 20 mL anhydrous toluene under N_2 protection. The mixture was stirred at 110 ^oC for overnight and cooled down to room temperature. The solvent was evaporated under reduced pressure. Following, the crude product was purified through column chromatography to get 2.55 g compound (Yield 53%). ^{1}H NMR (300 MHz, $CDCl_3$): δ = 7.81 (m, 6H), 7.77-7.72 (m, 3H), 7.66 (m, 6H), 3.87-3.72 (m, 2H), 3.31 (t, *J* = 6.4 Hz, 2H), 1.81 (m, 4H), 1.67-1.60 (m, 2H). ^{13}C NMR (75 MHz, $CDCl_3$): δ = 135.11, 135.07, 133.74, 133.60, 130.63, 130.47, 118.76, 117.62, 33.51, 32.00, 28.92, 23.08, 21.88. ^{31}P NMR (122 MHz, $CDCl_3$): δ = 24.28. ESI-MS: m/z calc'd for $C_{23}H_{25}BrP^+$ $[M-Br]^+$ 411.0877, found. 411.0877.

Synthesis of TPA-S-TPP. TPA-S-Q (192 mg, 0.4 mM) and compound 8 (147 mg, 0.3 mM) were added to 5 mL anhydrous 1,2-dichlorobenzene under N_2 atmosphere. The mixture was stirred at 130 ^oC for overnight. After the mixture cooled down to room temperature, and the crude product was purified through column chromatography to get 55 mg TPA-S-TPP (Yield 19%). ^{1}H NMR (300 MHz, MeOD): δ = 9.10 (d, *J* = 6.6 Hz, 1H), 8.69 (d, *J* = 8.6 Hz, 1H), 8.39 (d, *J* = 9.0 Hz, 1H), 8.17 (dt, *J* = 15.9, 7.7 Hz, 3H), 7.94-7.79 (m, 7H), 7.79-7.67 (m, 10H), 7.54-7.43 (m, 3H), 7.36-7.23 (m, 5H), 7.11-7.05 (m, 6H), 6.95 (d, *J* = 8.3 Hz, 2H), 4.90 (t, *J* = 7.4 Hz, 2H), 3.56-3.43 (m, 2H), 2.08 (br, 2H), 1.76 (br, 4H). ^{13}C NMR (75 MHz, MeOD): δ = 153.05, 149.83, 148.63, 147.03, 146.19, 139.24, 138.12, 136.45, 135.09, 134.89, 134.85, 133.56, 133.52, 133.43, 133.38, 130.24, 130.08, 129.28, 129.03, 126.65, 126.58, 126.43, 126.01, 124.90, 123.70, 123.62, 121.97, 119.15, 119.02, 118.65, 118.00, 117.87, 116.44, 115.19, 56.49, 28.76, 27.21, 21.87, 20.96. ESI-MS: m/z calc'd for $C_{56}H_{49}BrN_2PS^+$ $[M-Br]^+$ 891.2532, found. 891.2537.

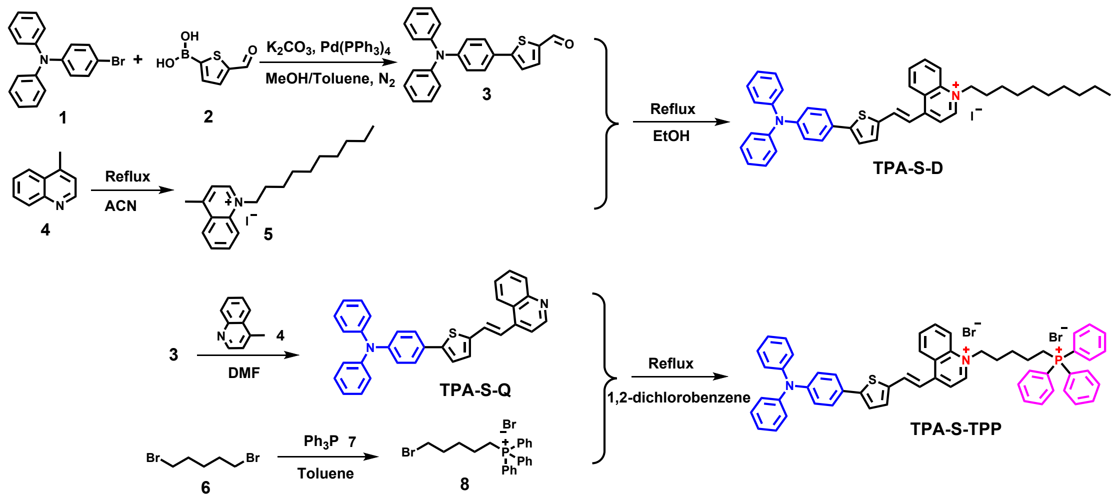

Fig. S1 Synthesis routes of TPA-S-Q, TPA-S-D, and TPA-S-TPP.

^{1}H-NMR, ^{13}C-NMR, ^{31}P-NMR, and MS spectra

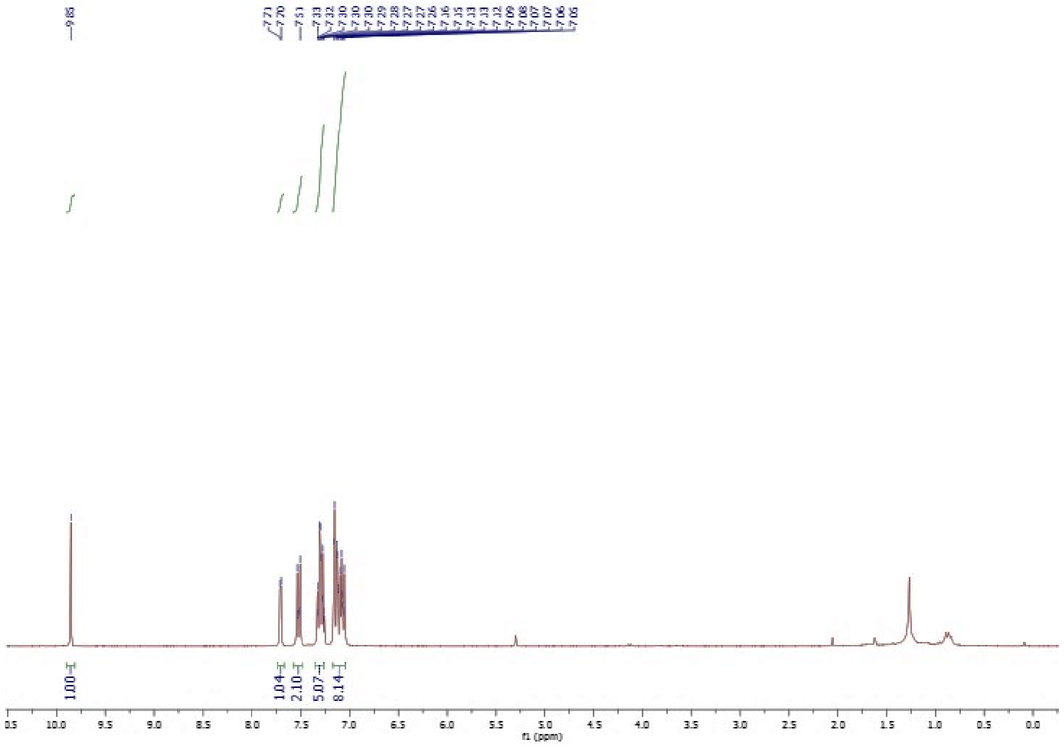

Fig. S2 ^{1}H NMR spectrum of compound 3.

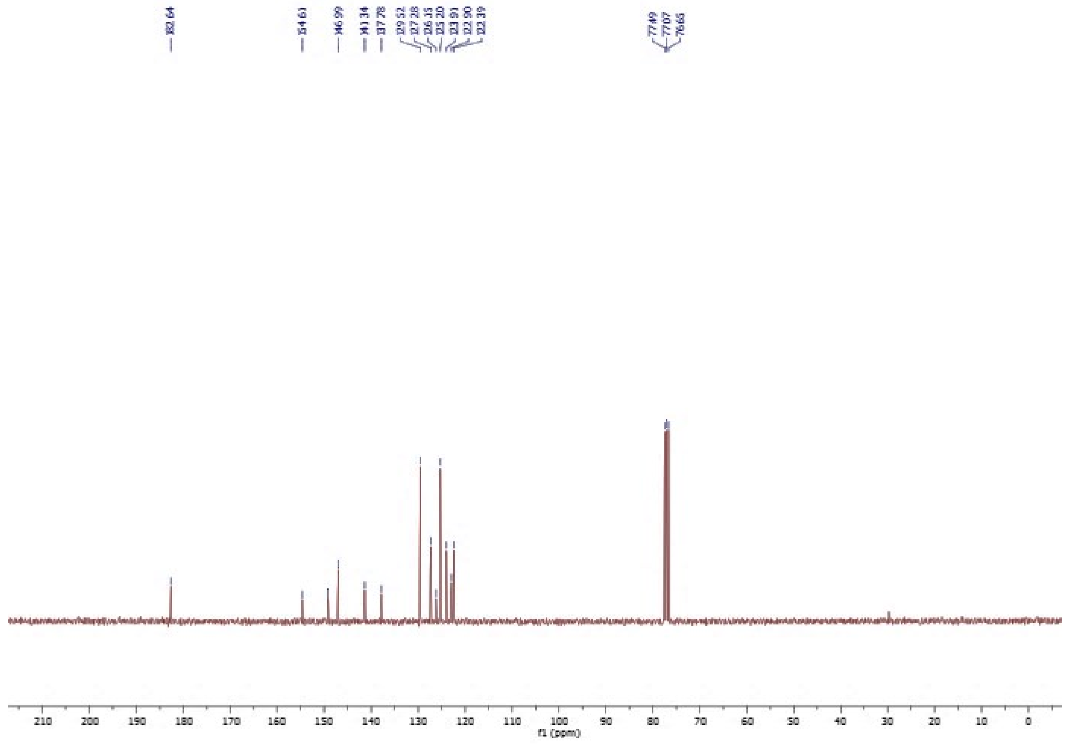

Fig. S3 ^{13}C NMR spectrum of compound 3.

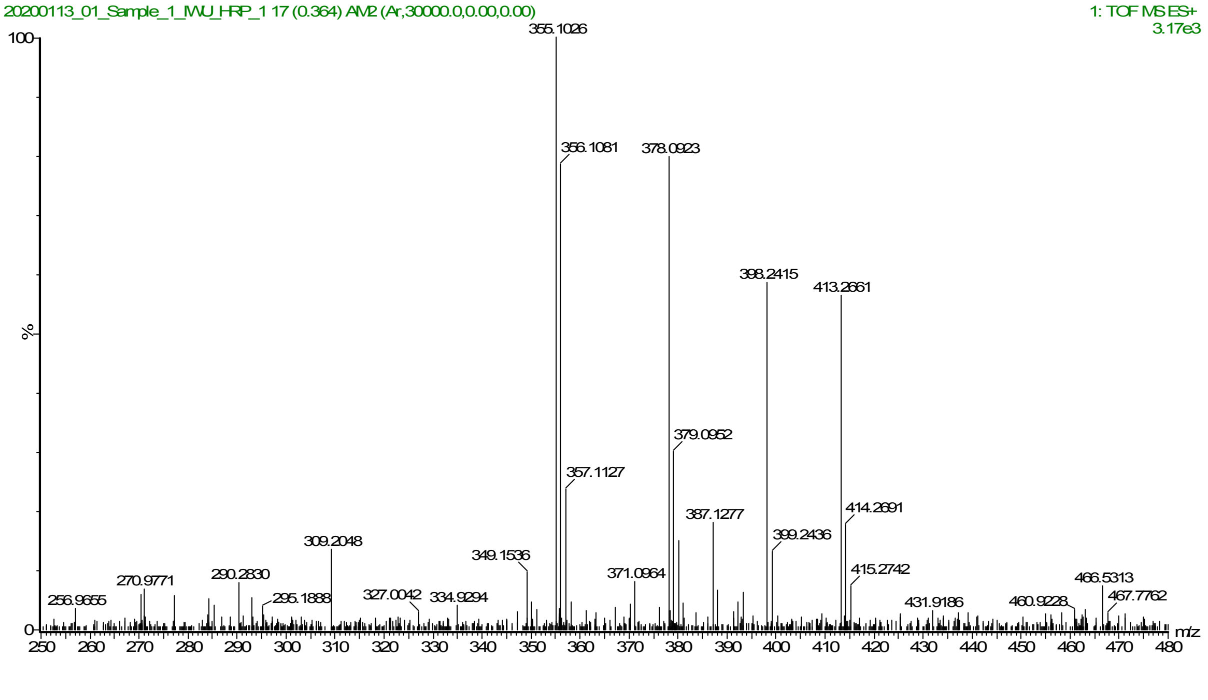

Fig. S4 ESI-HRMS of compound 3.

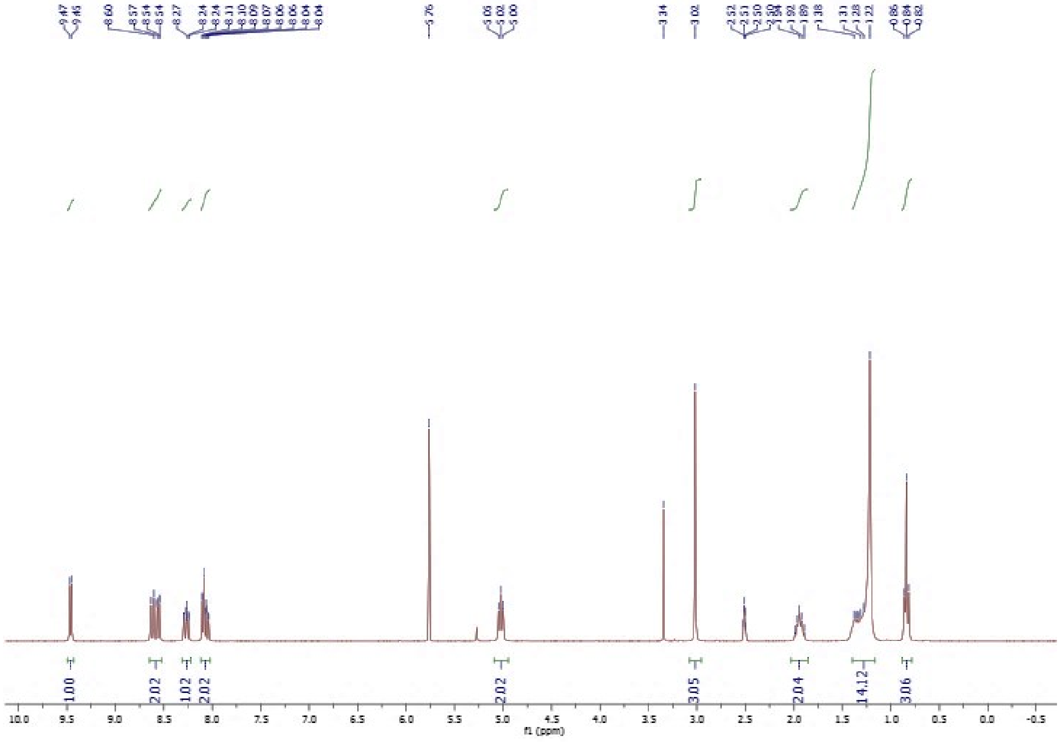

Fig. S5 ^{1}H NMR spectrum of compound 5.

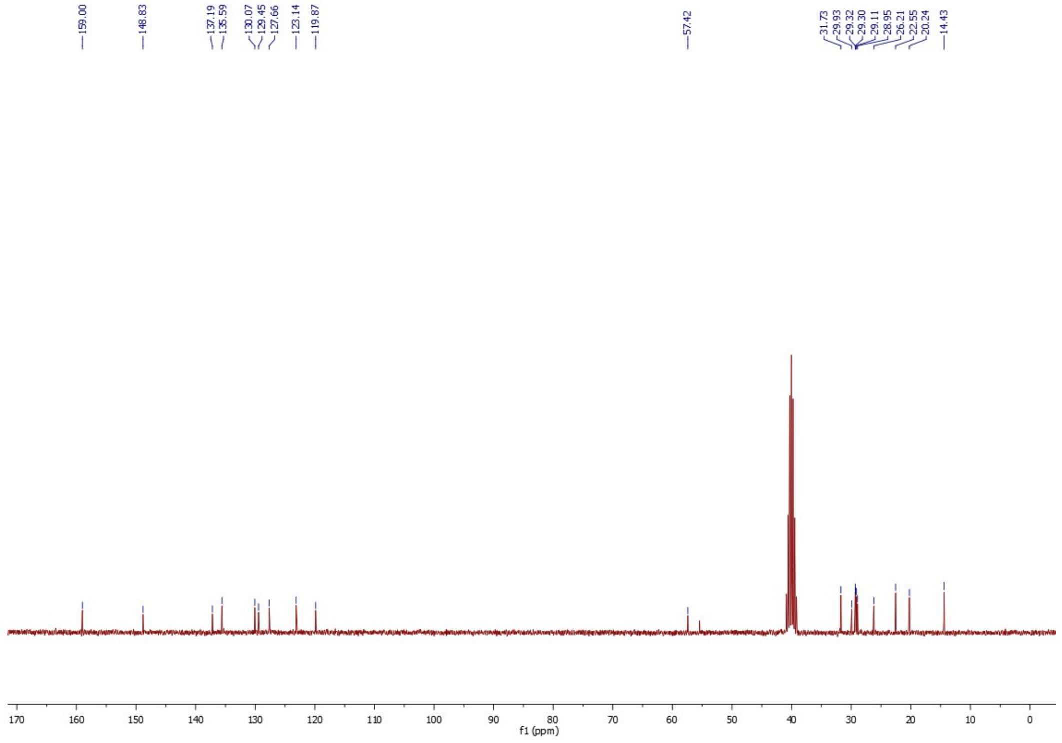

Fig. S6 ^{13}C NMR spectrum of compound 5.

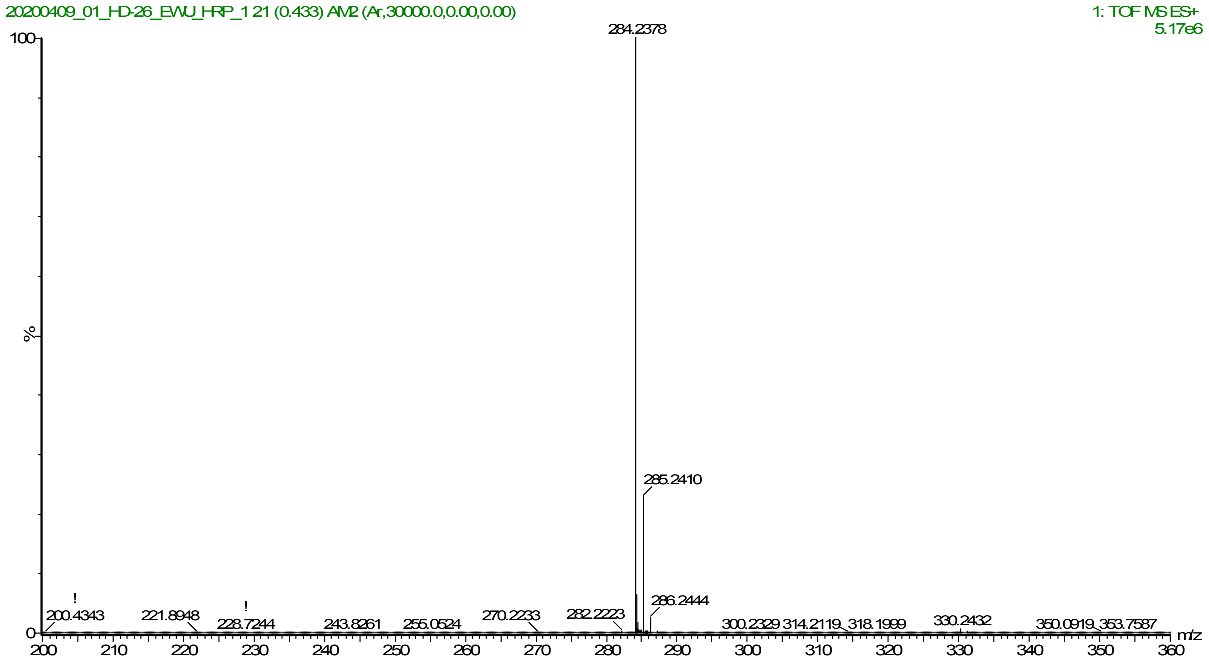

Fig. S7 ESI-HRMS of compound 5.

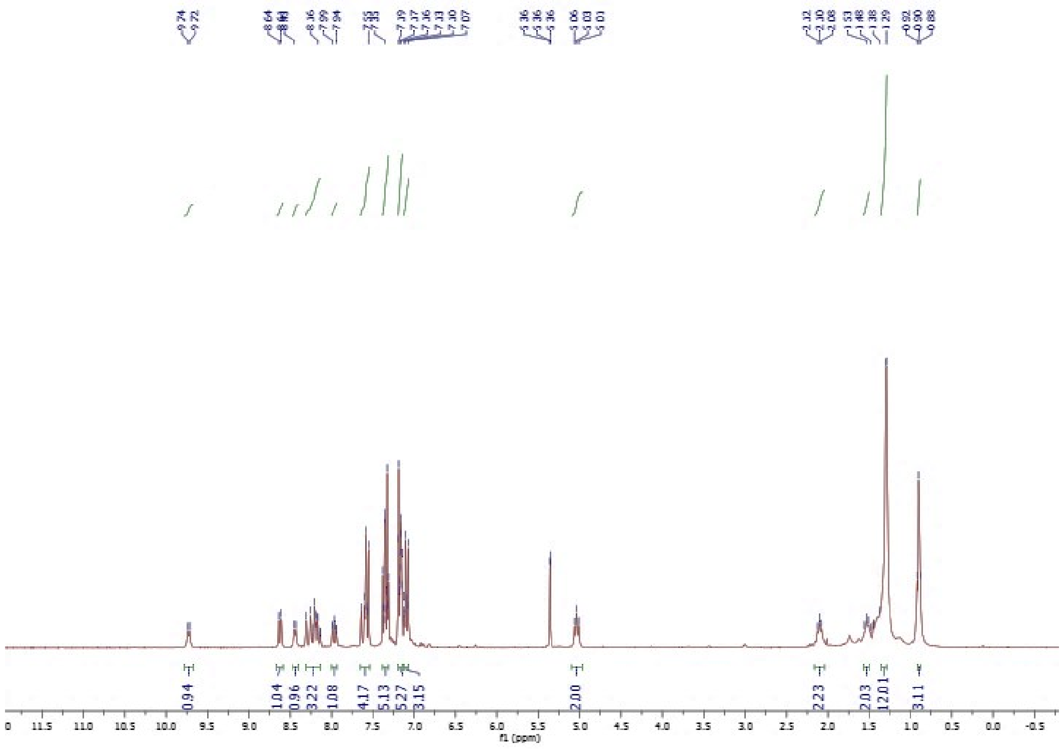

Fig. S8 ^{1}H NMR spectrum of TPA-S-D.

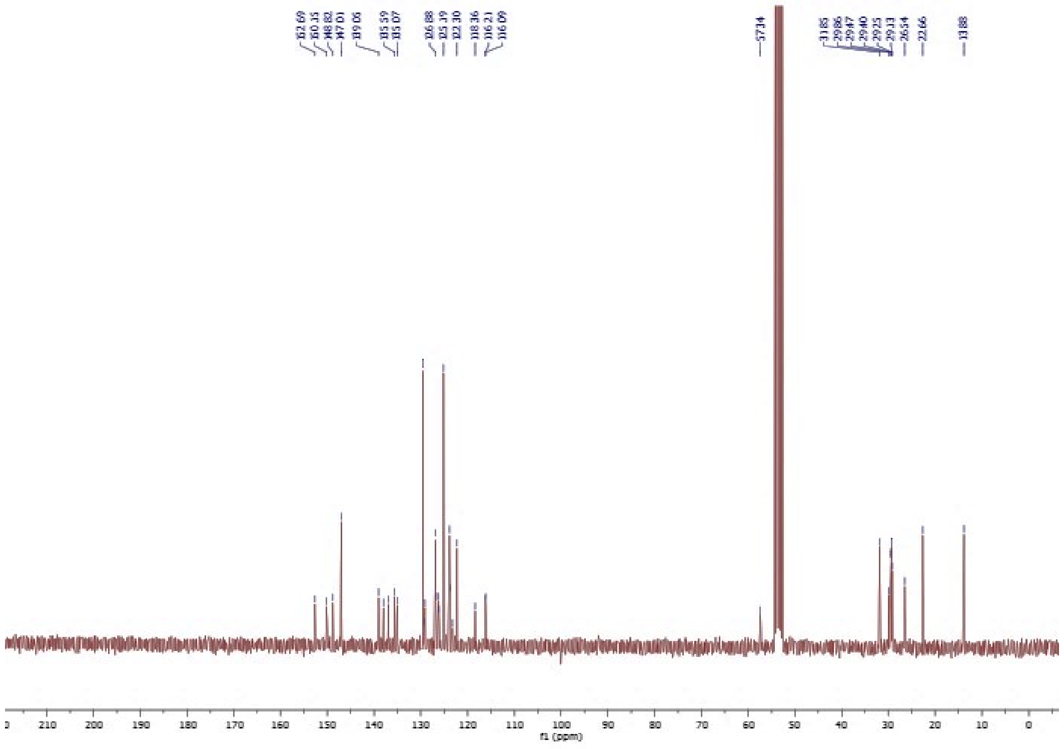

Fig. S9 ^{13}C NMR spectrum of TPA-S-D.

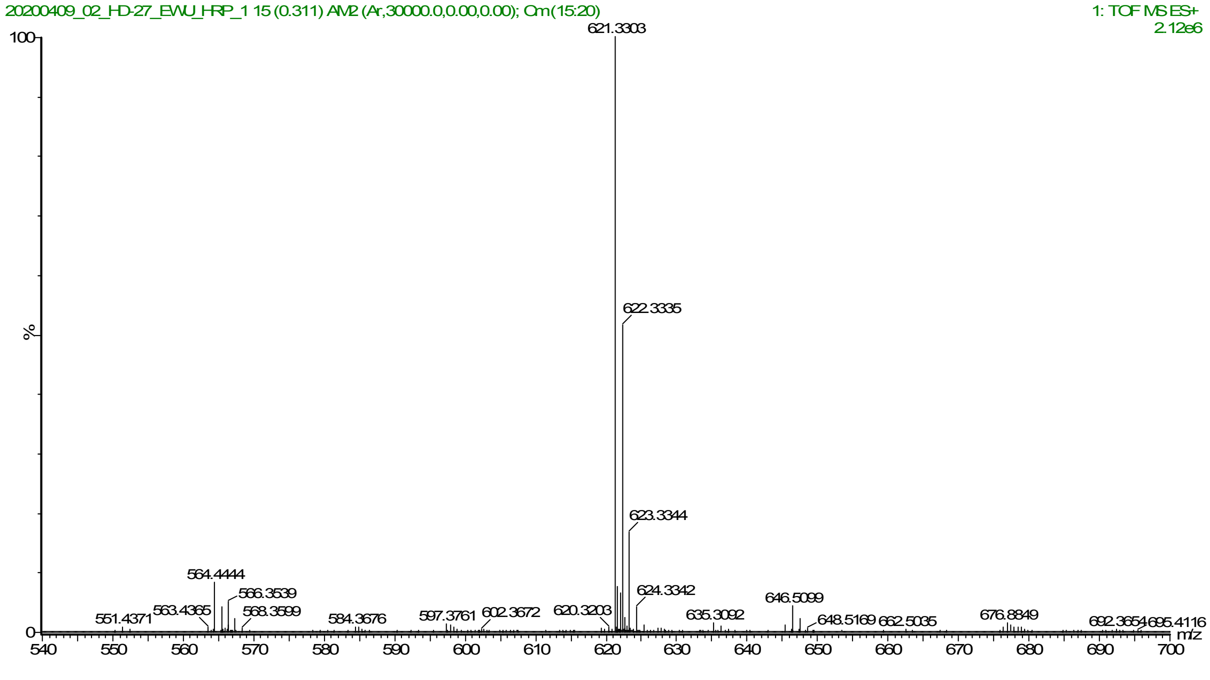

Fig. S10 ESI-HRMS of TPA-S-D.

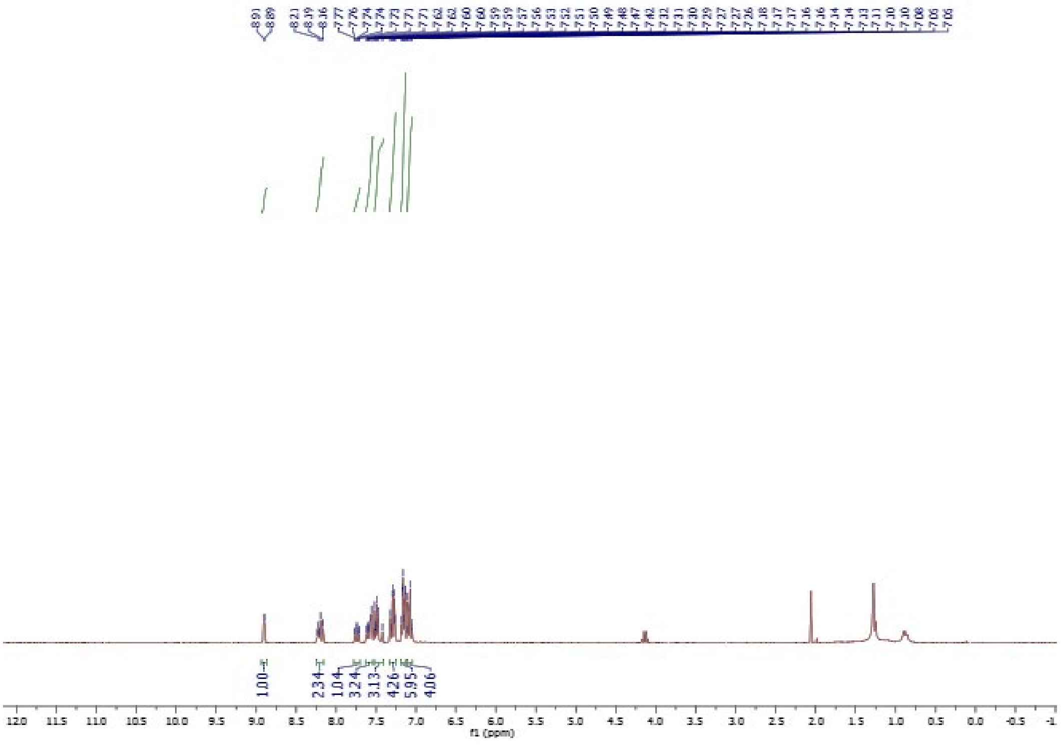

Fig. S11 ^{1}H NMR spectrum of TPA-S-Q.

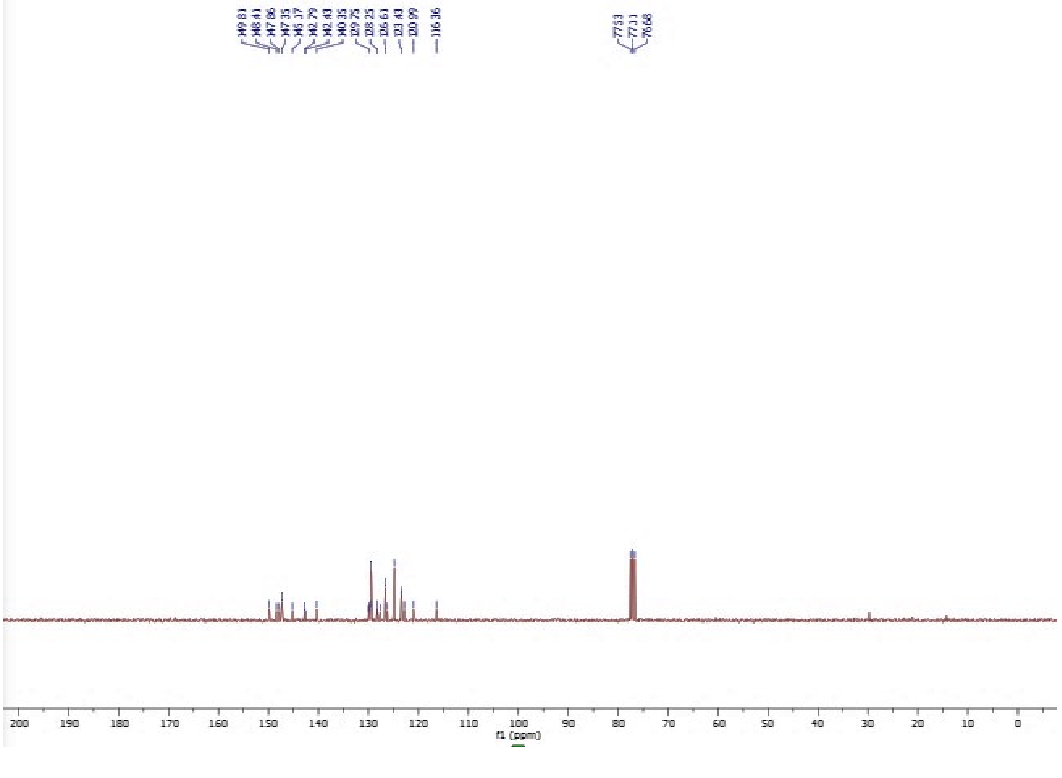

Fig. S12 ^{13}C NMR spectrum of TPA-S-Q.

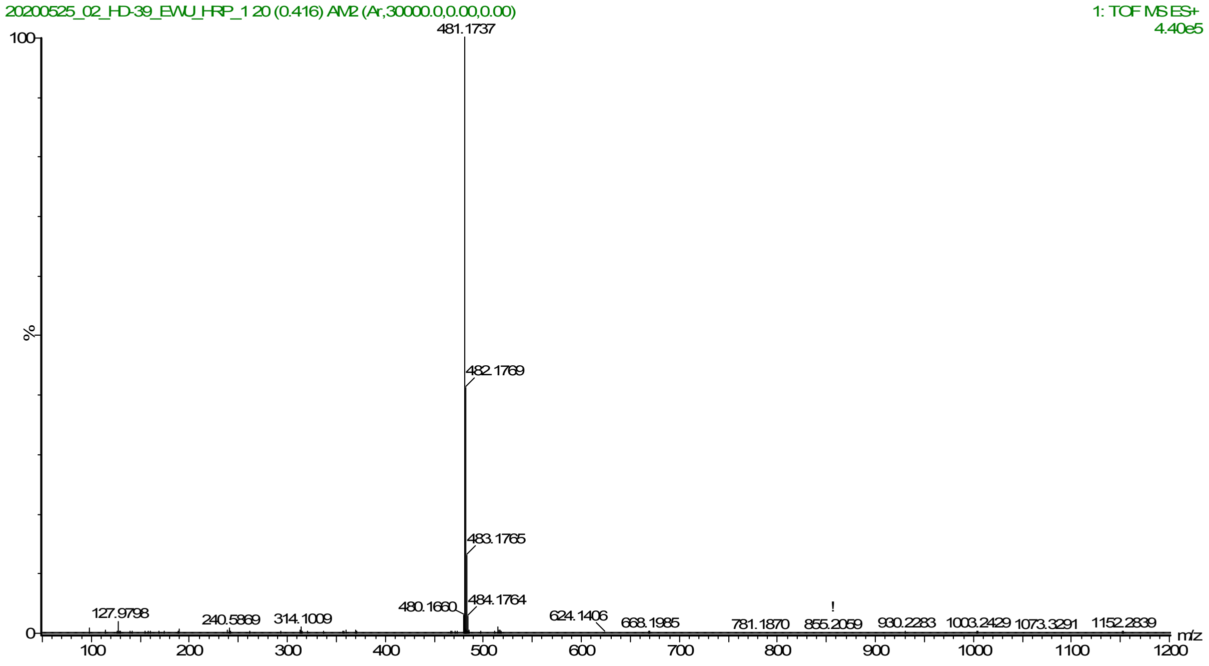

Fig. S13 ESI-HRMS of TPA-S-Q.

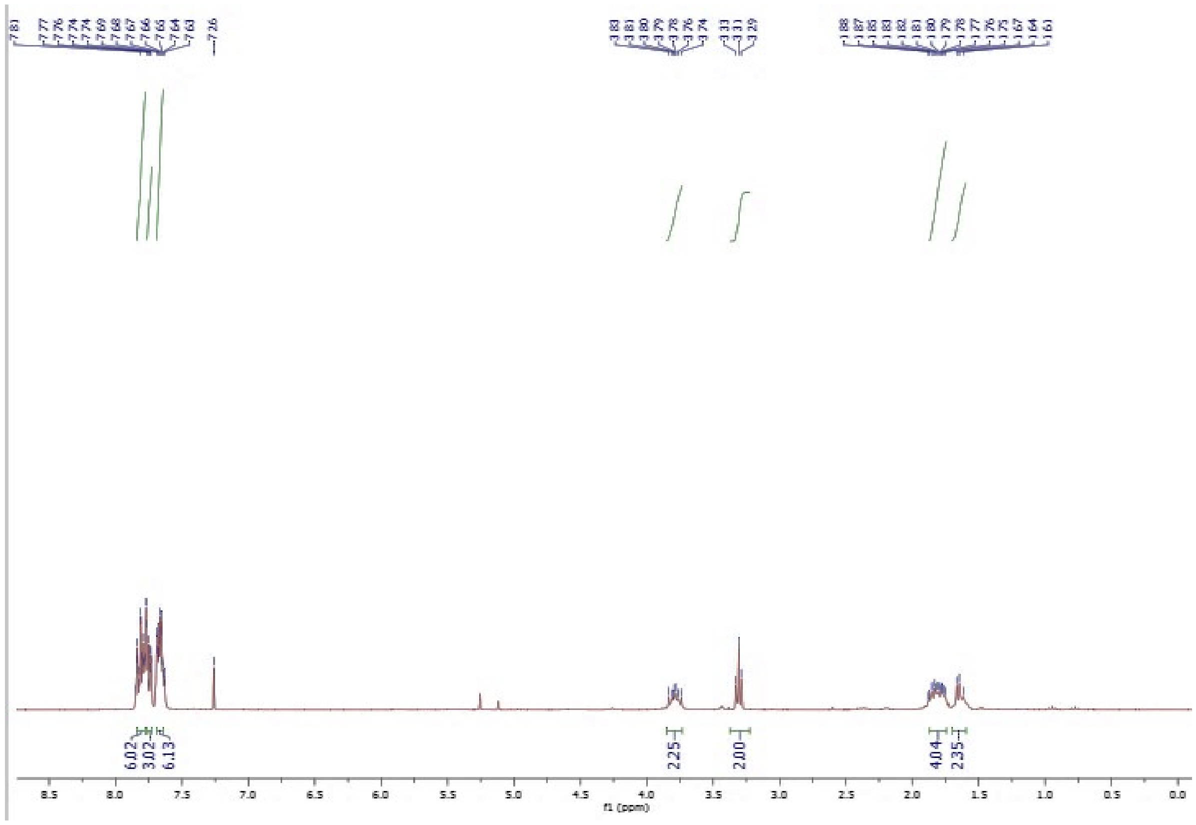

Fig. S14 ^{1}H NMR spectrum of compound 8.

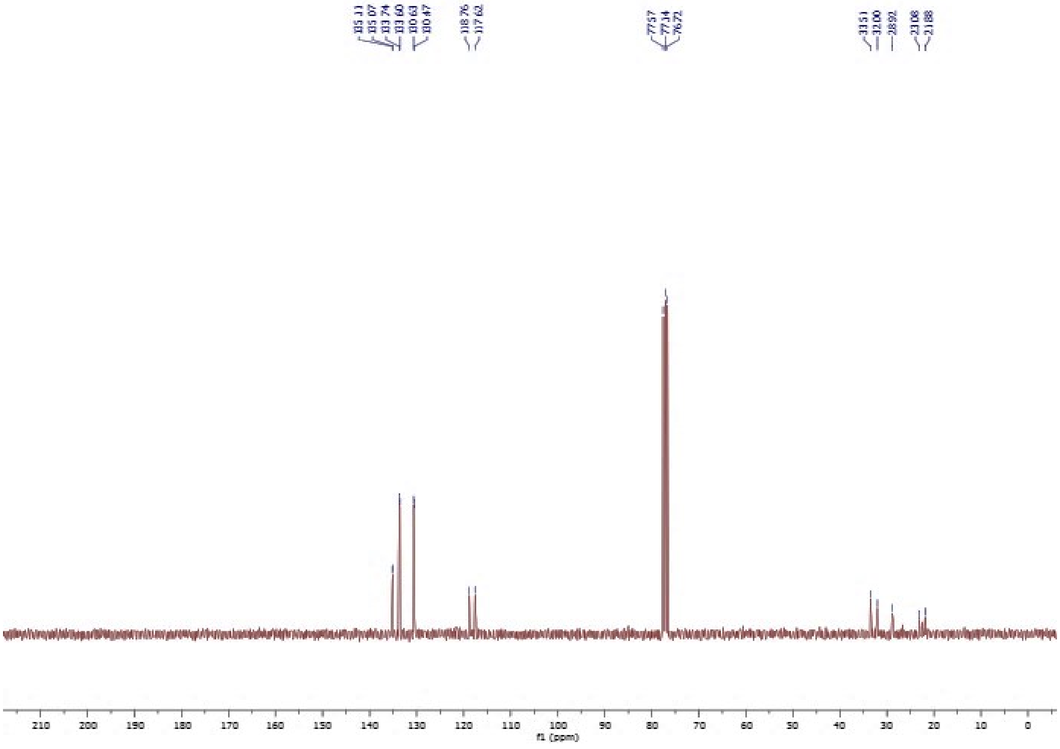

Fig. S15 ^{13}C NMR spectrum of compound 8.

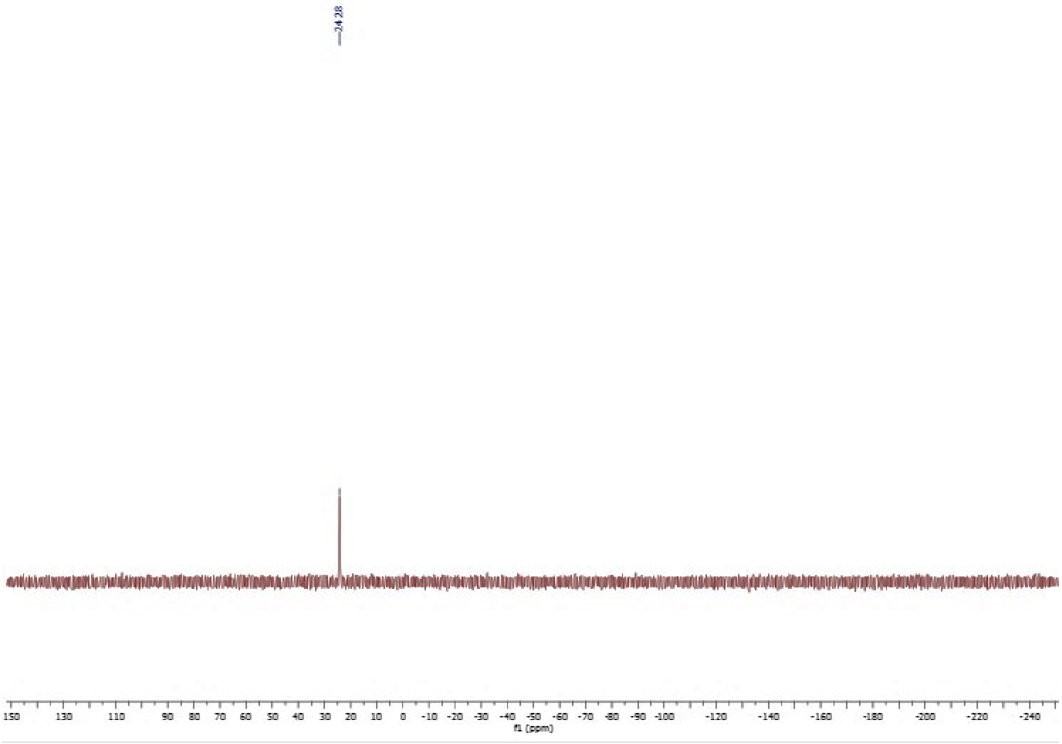

Fig. S16 ^{31}P NMR of compound 8.

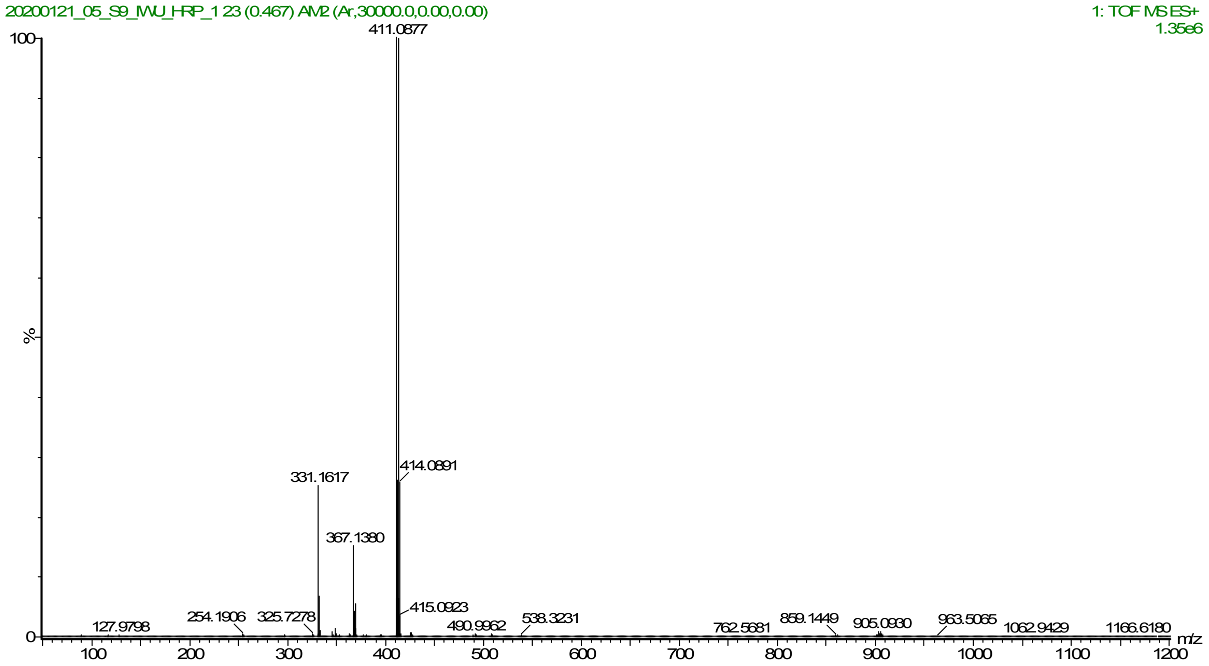

Fig. S17 ESI-HRMS of compound 8.

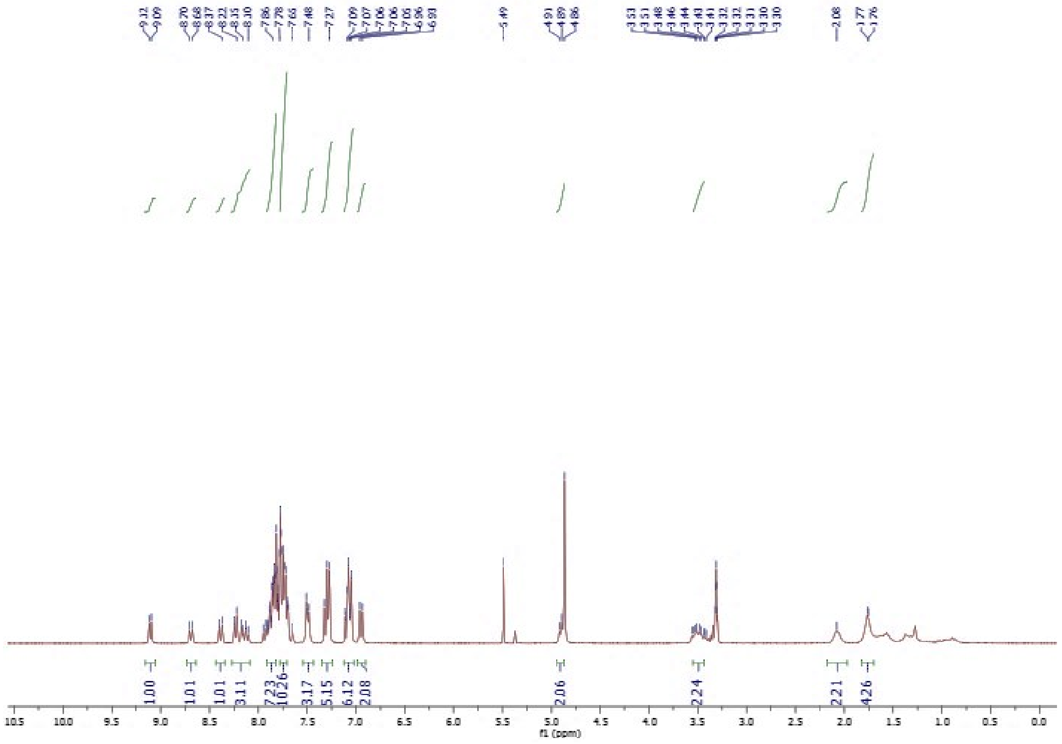

Fig. S18 ^{1}H NMR spectrum of TPA-S-TPP.

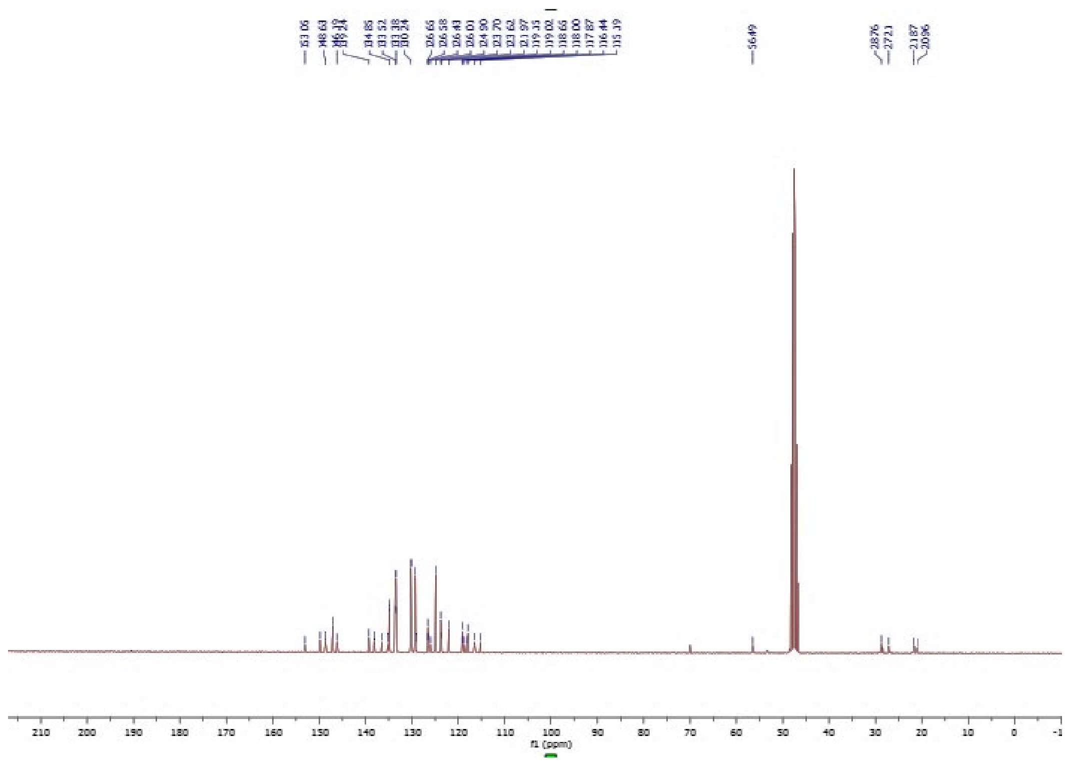

Fig. S19 ^{13}C NMR spectrum of TPA-S-TPP.

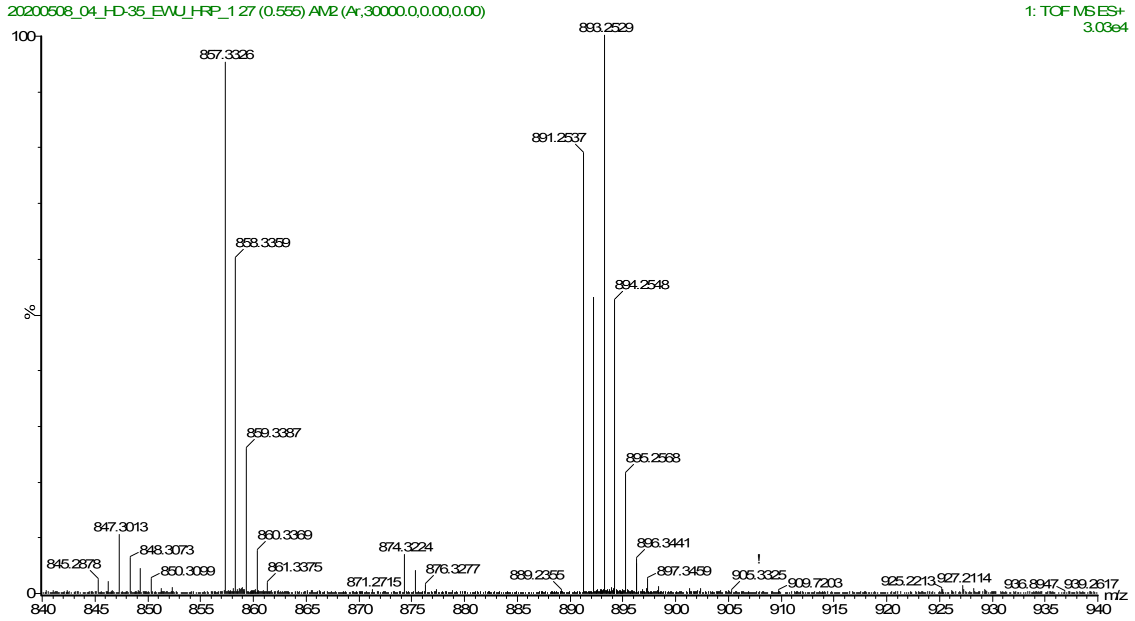

Fig. S20 ESI-HRMS of TPA-S-TPP.

Results and discussion

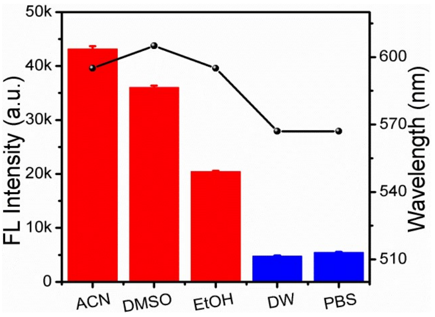

Fig. S21 Fluorescence intensity and maximum emission peak of TPA-S-Q (10 μM) in various solvents (ACN, DMSO, EtOH, DW, and PBS).

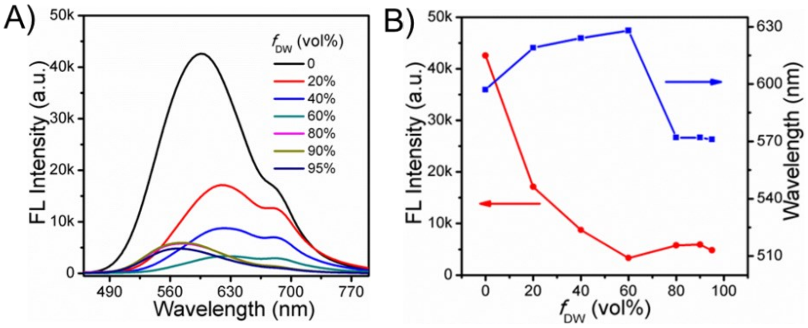

Fig. S22 A) Fluorescence spectra changes of TPA-S-Q (10 μM) in the ACN/DW (DW fraction f_{DW}: 0-95%) mixed solution system. B) Fluorescence intensity and maximum emission peak of TPA-S-Q (10 μM) in mixtures of ACN/DW (DW fraction f_{DW}: 0-95%).

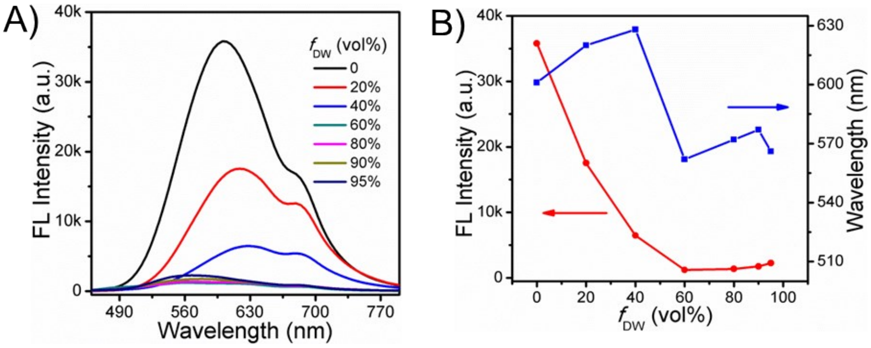

Fig. S23 A) Fluorescence spectra changes of TPA-S-Q (10 μM) in the DMSO/DW (DW fraction f_{DW}: 0-95%) mixed solution system. B) Fluorescence intensity and maximum emission peak of TPA-S-Q (10 μM) in mixtures of DMSO/DW (DW fraction f_{DW}: 0-95%).

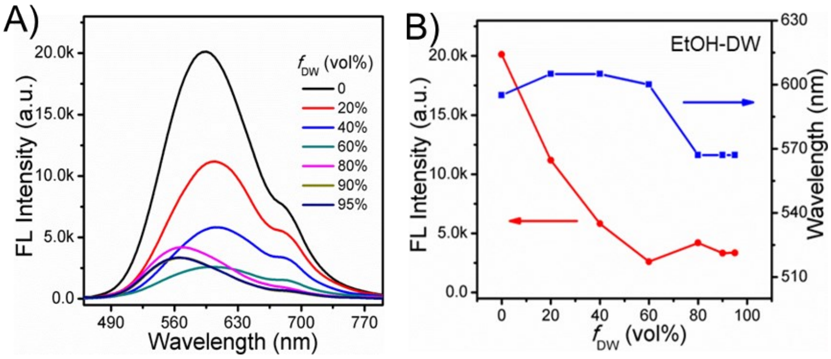

Fig. S24 A) Fluorescence spectra changes of TPA-S-Q (10 μM) in the EtOH/DW (DW fraction f_{DW}: 0-95%) mixed solution system. B) Fluorescence intensity and maximum emission peak of TPA-S-Q (10 μM) in mixtures of EtOH/DW (DW fraction f_{DW}: 0-95%).

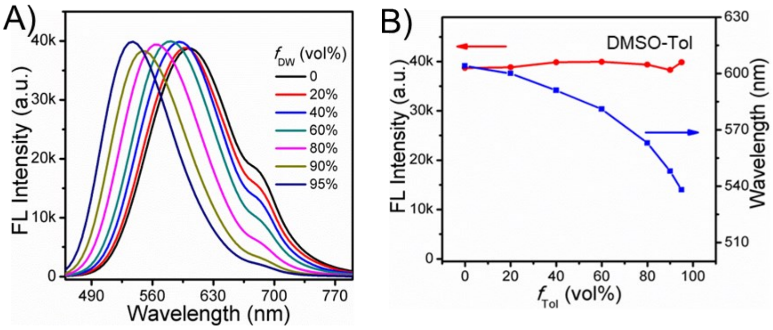

Fig. S25 A) Fluorescence spectra changes of TPA-S-Q (10 μM) in the DMSO/Tol (Toluene fraction f_{Tol}: 0-95%) mixed solution system. B) Fluorescence intensity and maximum emission peak of TPA-S-Q (10 μM) in mixtures of DMSO/Tol (Toluene fraction f_{Tol}: 0-95%).

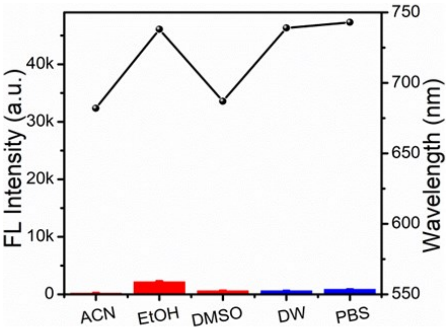

Fig. S26 Fluorescence intensity and maximum emission peak of TPA-S-D (10 μM) in various solvents (ACN, EtOH, DMSO, DW, and PBS).

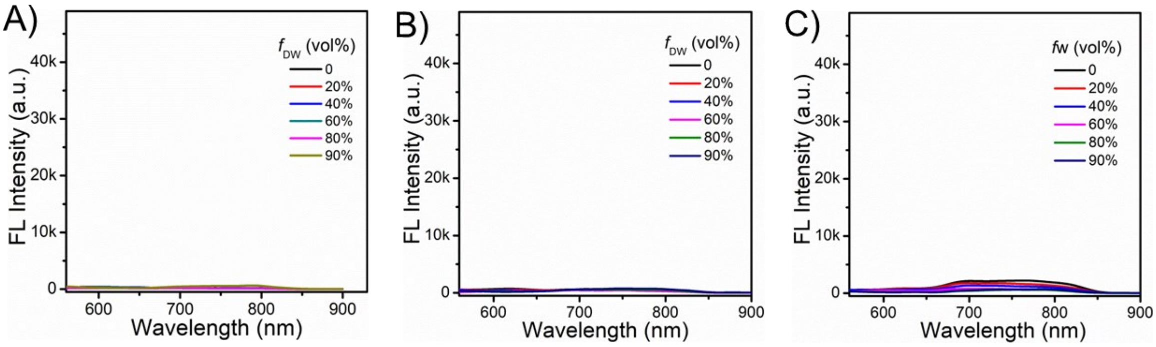

Fig. S27 A) Fluorescence spectra changes of TPA-S-D (10 μM) in the ACN/DW (A, DW fraction f_{DW}: 0-90%), DMSO/DW (B, DW fraction f_W: 0-90%), EtOH/DW (C, DW fraction f_{DW}: 0-90%) mixed solution system.

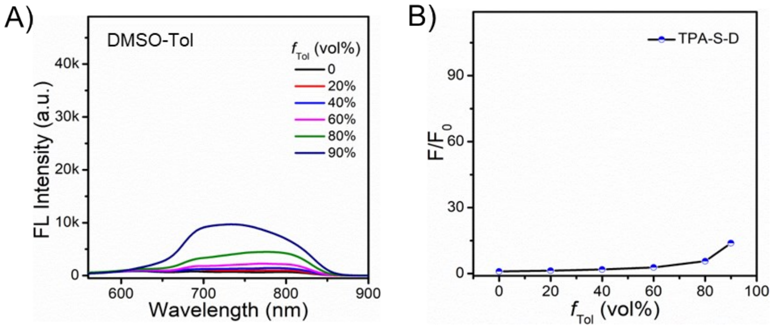

Fig. S28 A) Fluorescence spectra and plots of maximum fluorescence intensity changes (B) of TPA-S-D (10 μM) in the DMSO/toluene mixed solution system (toluene fraction f_{Tol}: 0-90%).

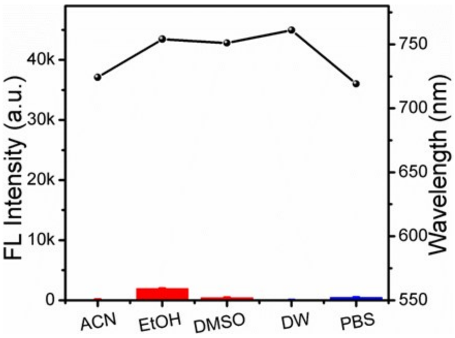

Fig. S29 Fluorescence intensity and maximum emission peak of TPA-S-TPP (10 μM) in various solvents (ACN, EtOH, DMSO, DW, and PBS).

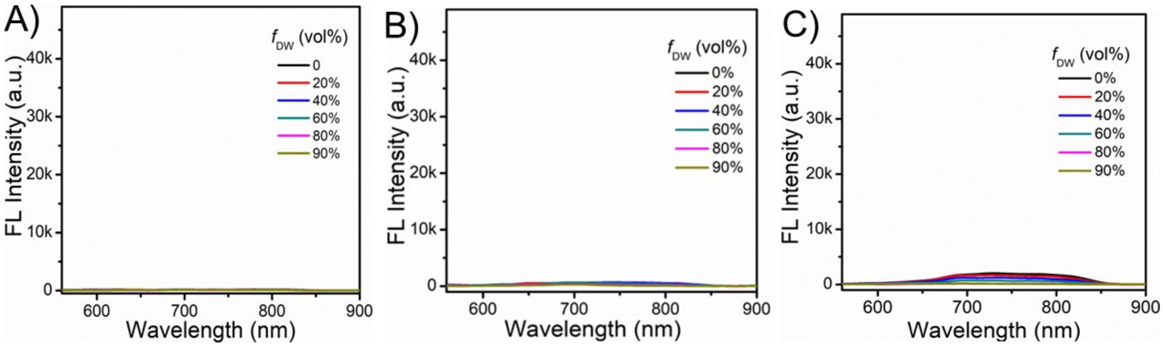

Fig. S30 Fluorescence spectra changes of TPA-S-TPP (10 μM) in the ACN/DW (A, DW fraction f_W: 0-90%), DMSO/DW (B, DW fraction f_{DW}: 0-90%), EtOH/DW (C, DW fraction f_{DW}: 0-90%) mixed solution system.

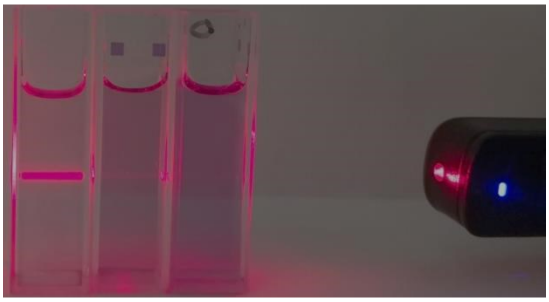

Fig. S31 Tyndall effect experiments of TPA-S-TPP (10 μM) in different solvents (from left to right: Toluene, DW, and DMSO) using a red pointer pen.

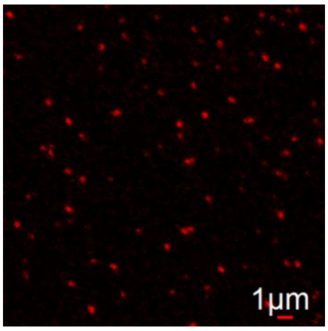

Fig. S32 Fluorescence imaging of TPA-S-TPP (10 μM) in Toluene was collected under the excitation of 559 nm and the emission of 655-755 nm.

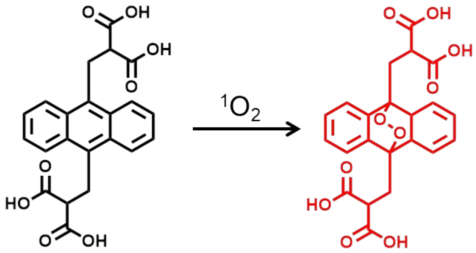

Fig. S33 Decomposition process of ABDA caused by $^1O_2$.

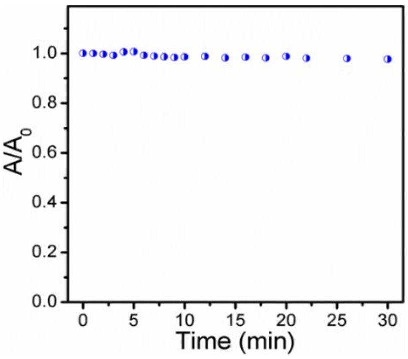

Fig. S34 Stability of AIEgen TPA-S-TPP (10 μM) under white light irrdiation (25 mW/cm^2) for different time (0-30 min).

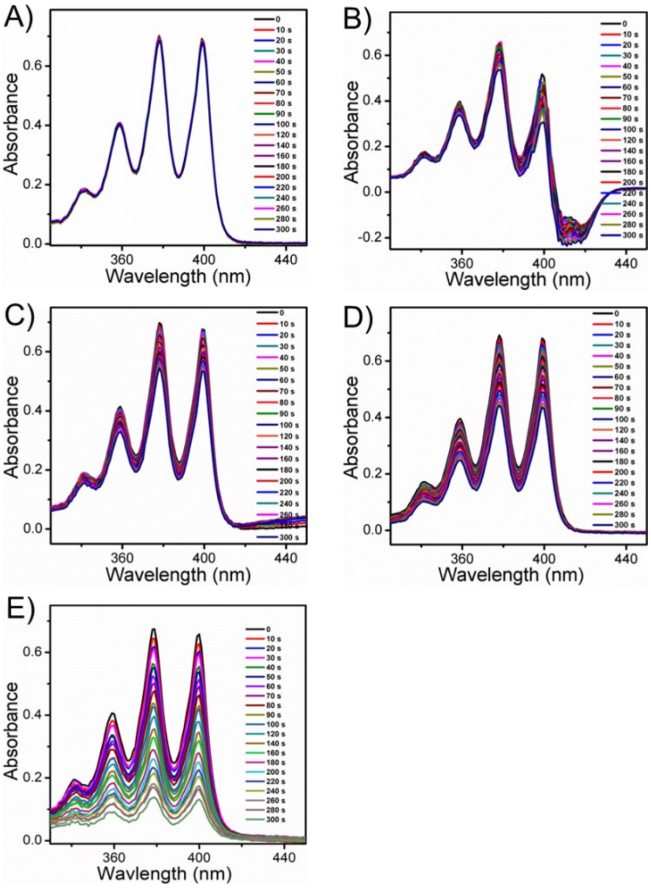

Fig. S35 Absorption spectra changes of A: ABDA (50 μM), B: ABDA (50 μM) + Ce6 (10 μM), C: ABDA (50 μM) + TPA-S-Q (10 μM), D: ABDA (50 μM) + Rose Bengal (10 μM), E: ABDA (50 μM) + TPA-S-D (10 μM) under white light irrdiation (25 mW/cm^2) for different time (0-300 s).

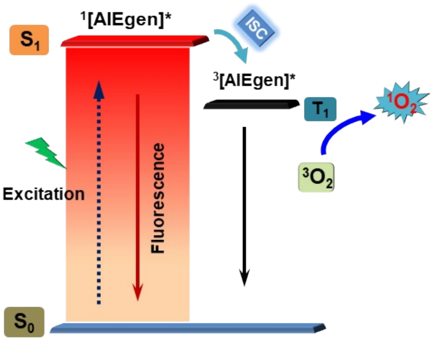

Fig. S36 Mechanism of $^1O_2$ generation with light irradiation.

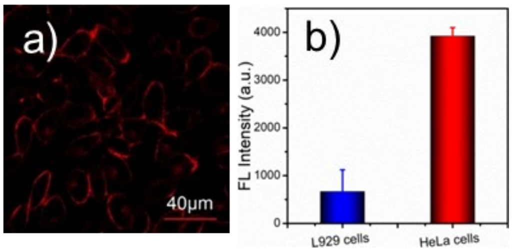

Fig. S37 Confocal imaging of L929 cells (normal cells) treated with TPA-S-TPP (5 μM) for 2 h. a) Fluorescence imaging, b) fluorescence intensities were recorded from L929 cells and HeLa cells. λex = 559 nm and λem = 655-755 nm. Scale bar = 40 μm.

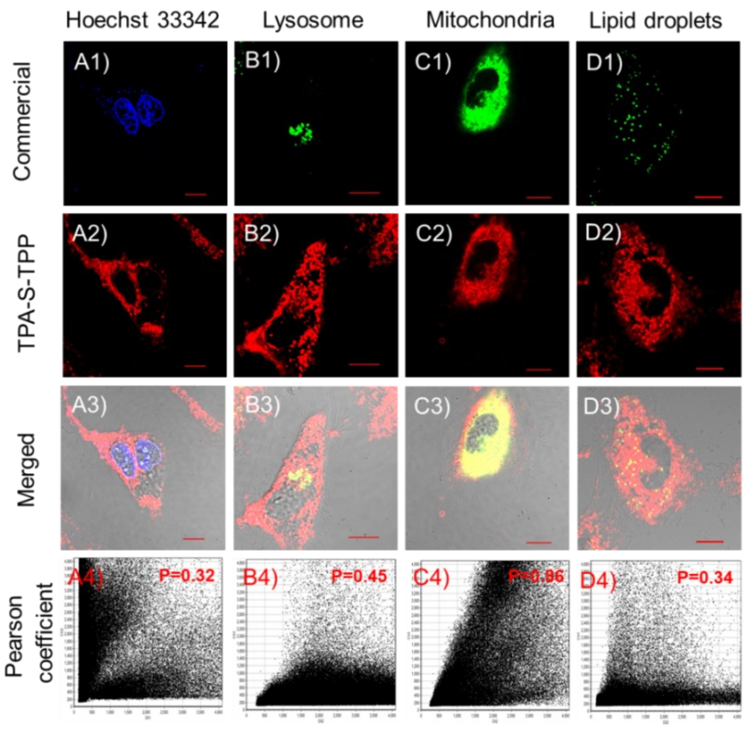

Fig. S38 Subcellular co-localization images of AIEgen TPA-S-TPP (A2, B2, C2, and D2) with commercial dyes Hoechst 33342 (A1), Lyso-Tracker Green (B1), Mito-Tracker Green (C1), and BODIPY 493/503 (D1) under the direction of the instructions, respectively. A3), B3), C3), and D3) Merged images. Pearson's coefficient: A4, B4, C4, and D4. Scale bar = 10 µm.

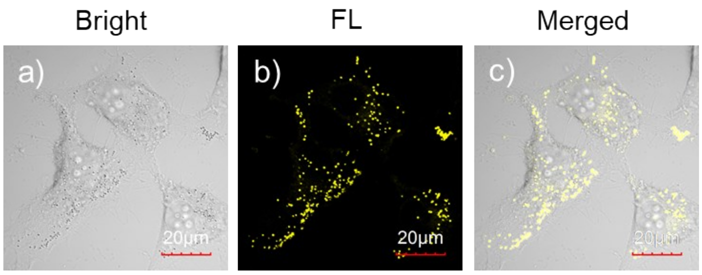

Fig. S39 Fluorescence imaging of HeLa cells treated with TPA-S-Q. Scale bar = 20 µm.

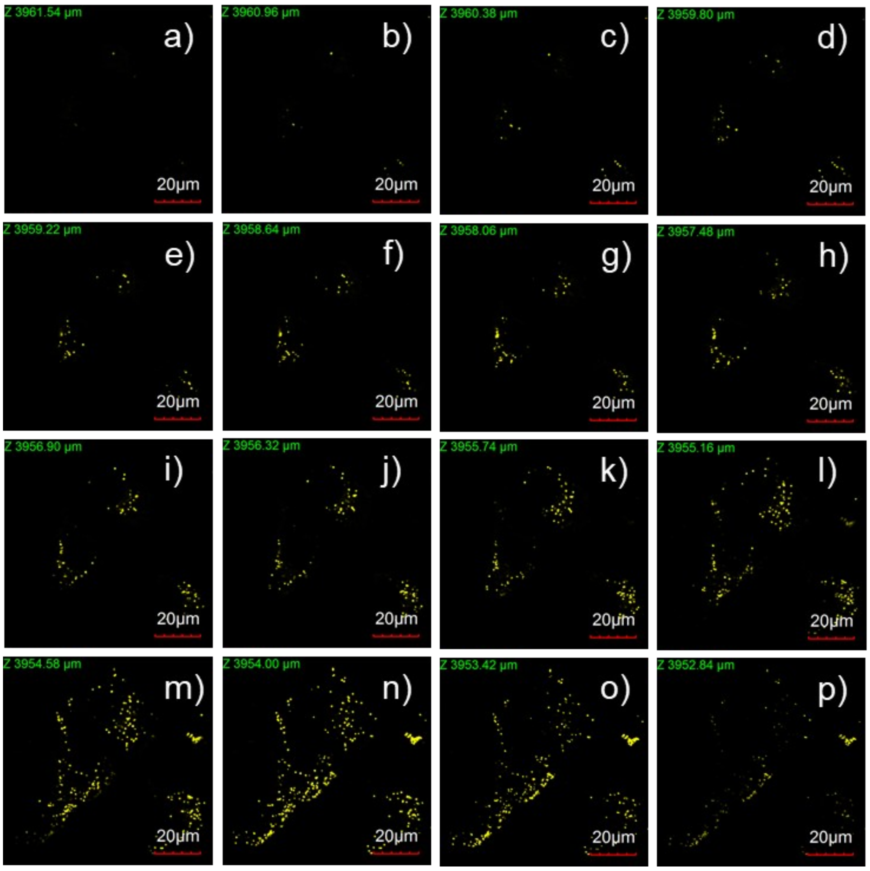

Fig. S40 3D imaging of HeLa cells along the longitudinal *Z* axis (based on **Fig. S39**, from 3961.54 μm to 3952.84 μm, step size = 0.58 μm) treated with TPA-S-Q under the excitation of 405 nm (emission collected 490-540 nm). Scale bar = 20 μm.

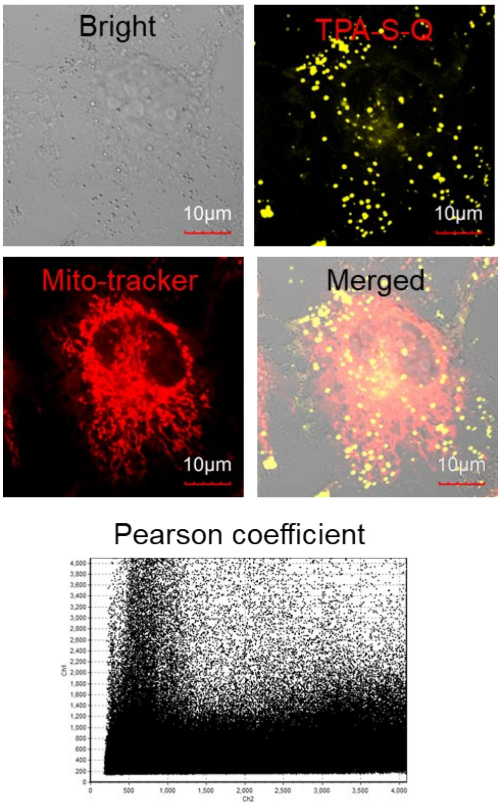

Fig. S41 Confocal fluorescence images of HeLa cells incubated with TPA-S-Q and Mito-tracker deep red.

Scale bar = 10 μm.

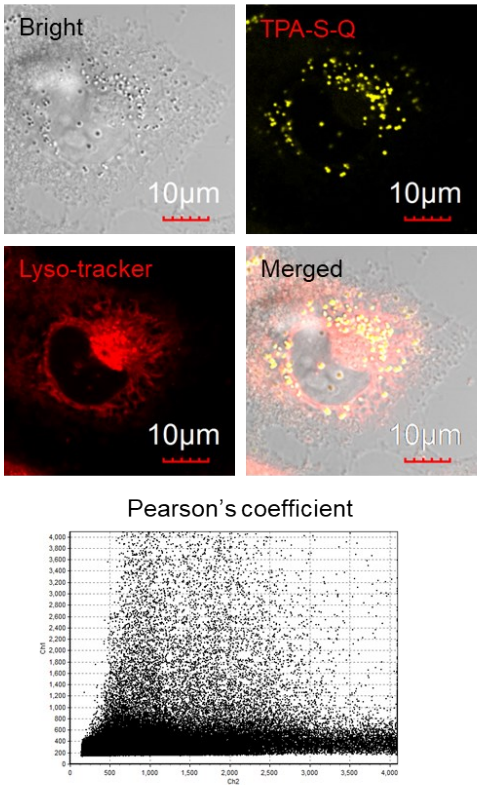

Fig. S42 Confocal fluorescence images of HeLa cells incubated with TPA-S-Q and Lyso-Tracker Deep Red. Scale bar = 10 μm.

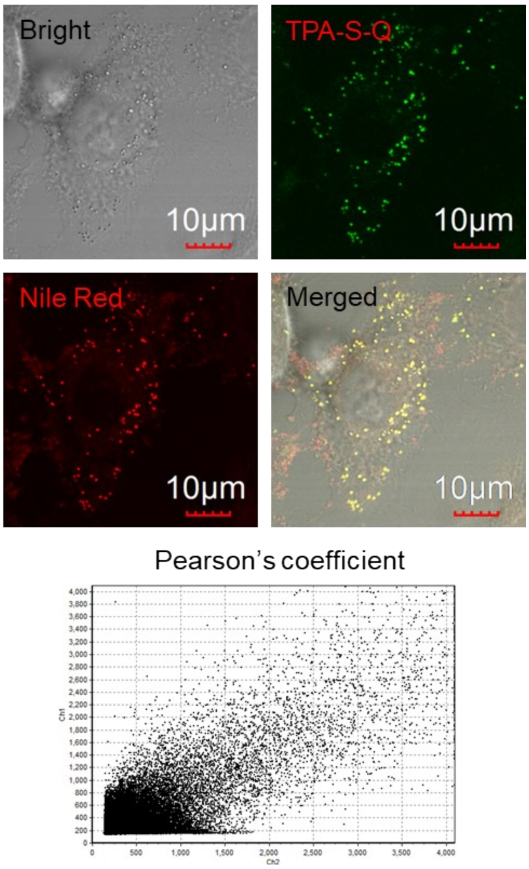

Fig. S43 Confocal fluorescence images of HeLa cells incubated with TPA-S-Q and Nile Red. Scale bar = 10 μm.

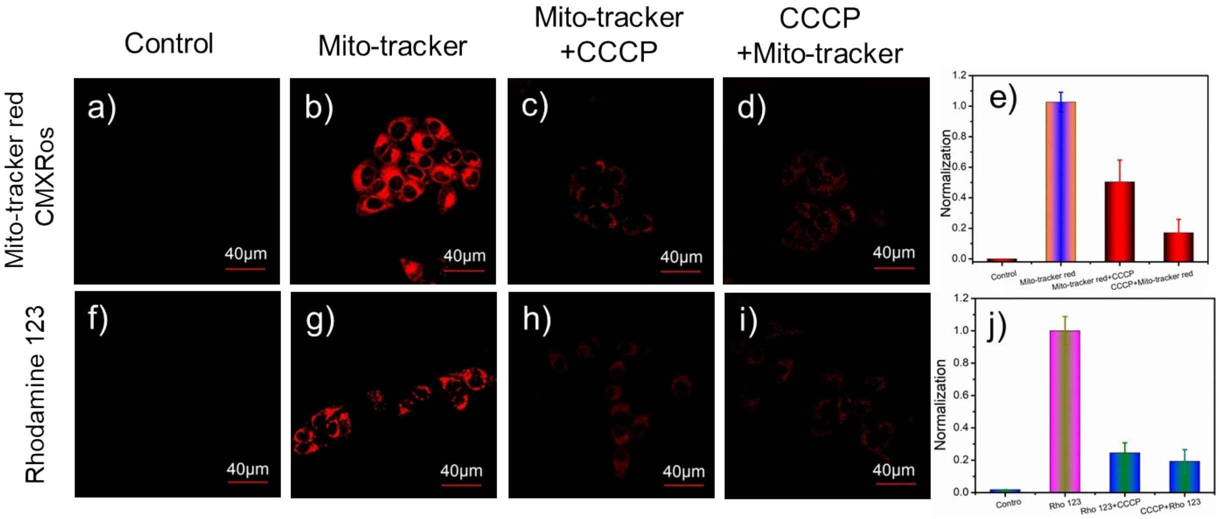

Fig. S44 Confocal fluorescence images of HeLa cells to investigate the influence of mitochondrial membrane potential by CCCP. Mito-tracker red CMXRos: a) control group; b) Only treated with Mito-Tracker Red; c) pre-incubated with Mito-Tracker Red and then added CCCP (10 μM, 20 min); d) pre-incubated with CCCP (10 μM, 20 min) and then added Mito-Tracker Red. e) Normalized fluorescence intensities based on the results of HeLa cell imaging (a-d). Note: the pixel intensity of Mito-Tracker Red (b) was defined as 1.0. Rhodamine 123: f) control group; g) treated with Rho 123; h) pre-incubated with Rho 123 and then added CCCP (10 μM, 20 min); i) pre-incubated with CCCP (10 μM, 20 min) and then added Rho 123. j) Normalized fluorescence intensities based on the results of HeLa cell imaging (a-g). Note: the pixel intensity of Rho 123 (g) was defined as 1.0. Scale bar = 40 μm.

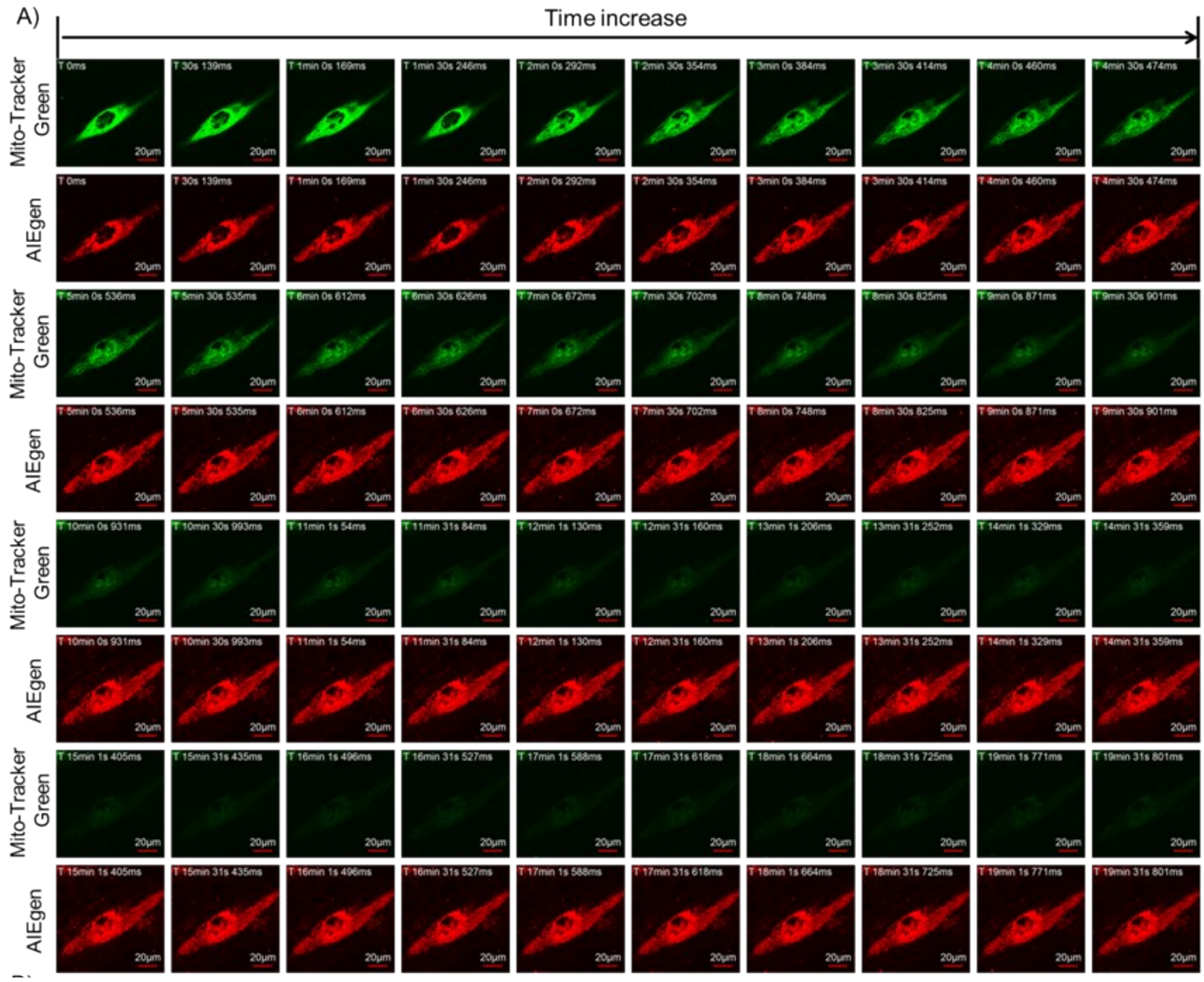

Fig. S45 A) Confocal fluorescence images of HeLa cells treated with Mito-Tracker Green and AIEgen TPA-S-TPP under continuous laser irradiation. Scale bar = 20 µm.

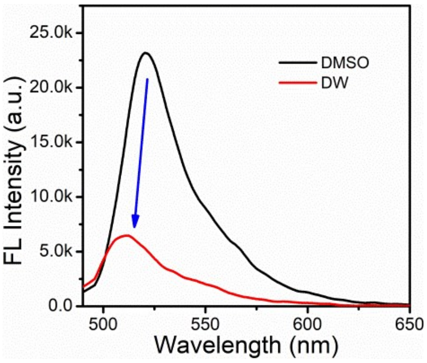

Fig. S46 Fluorescence spectra of Mito-tracker green (5 µm) in DMSO and DW.

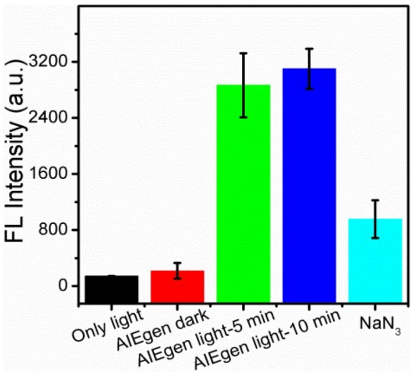

Fig. S47 Fluorescence intensities recorded from ROS generation in HeLa cells after different treatments using commercial DCF-DA dye.

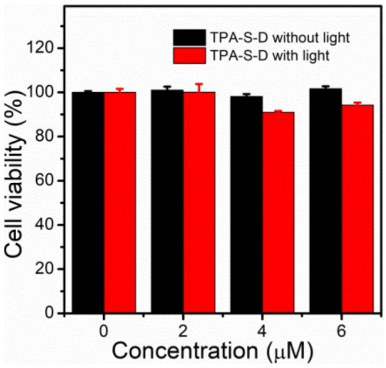

Fig. S48 MTT assays of HeLa cells treated with the various concentrations of TPA-S-D (0, 2, 4, and 6 μM) under dark and white light irradiation.

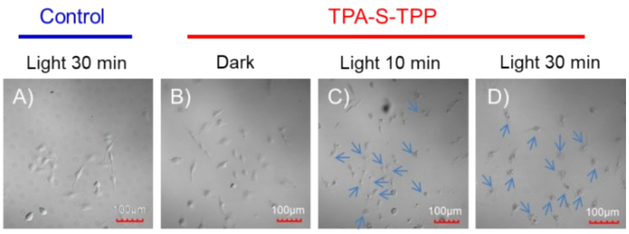

Fig. S49 Bright images of HeLa cells co-stained by Annexin V-FITC and propidium iodide (PI) dyes after different treatments: A, only light for 30 min; B, TPA-S-TPP + dark; C, TPA-S-TPP + light for 10 min; D, TPA-S-TPP + light for 30 min. Scale bar = 100 μm.

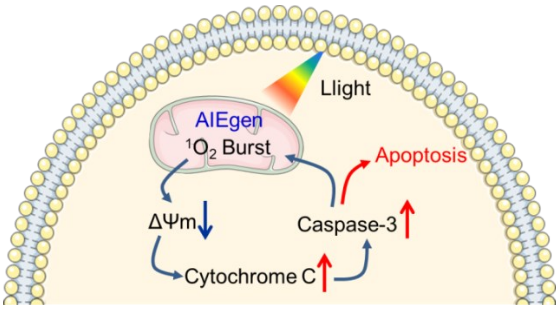

Fig. S50 Schematic of TPA-S-TPP for enhanced PDT in cells under light irradiation.
